# Supplementary material for: Differentiation of Hebbian and homeostatic plasticity mechanisms within layer 5 visual cortex neurons
Source: Cell Rep. 2022 May 31;39(9):110892. doi: 10.1016/j.celrep.2022.110892 (PMC9637998; doi:10.1016/j.celrep.2022.110892)
Supplement: Document Figures S1–S6 and Tables S1 and S2 [file mmc1.pdf]

Cell Reports, Volume *39*

Supplemental information

Differentiation of Hebbian and homeostatic plasticity mechanisms within layer 5 visual cortex neurons

Anurag Pandey, Neil Hardingham, and Kevin Fox

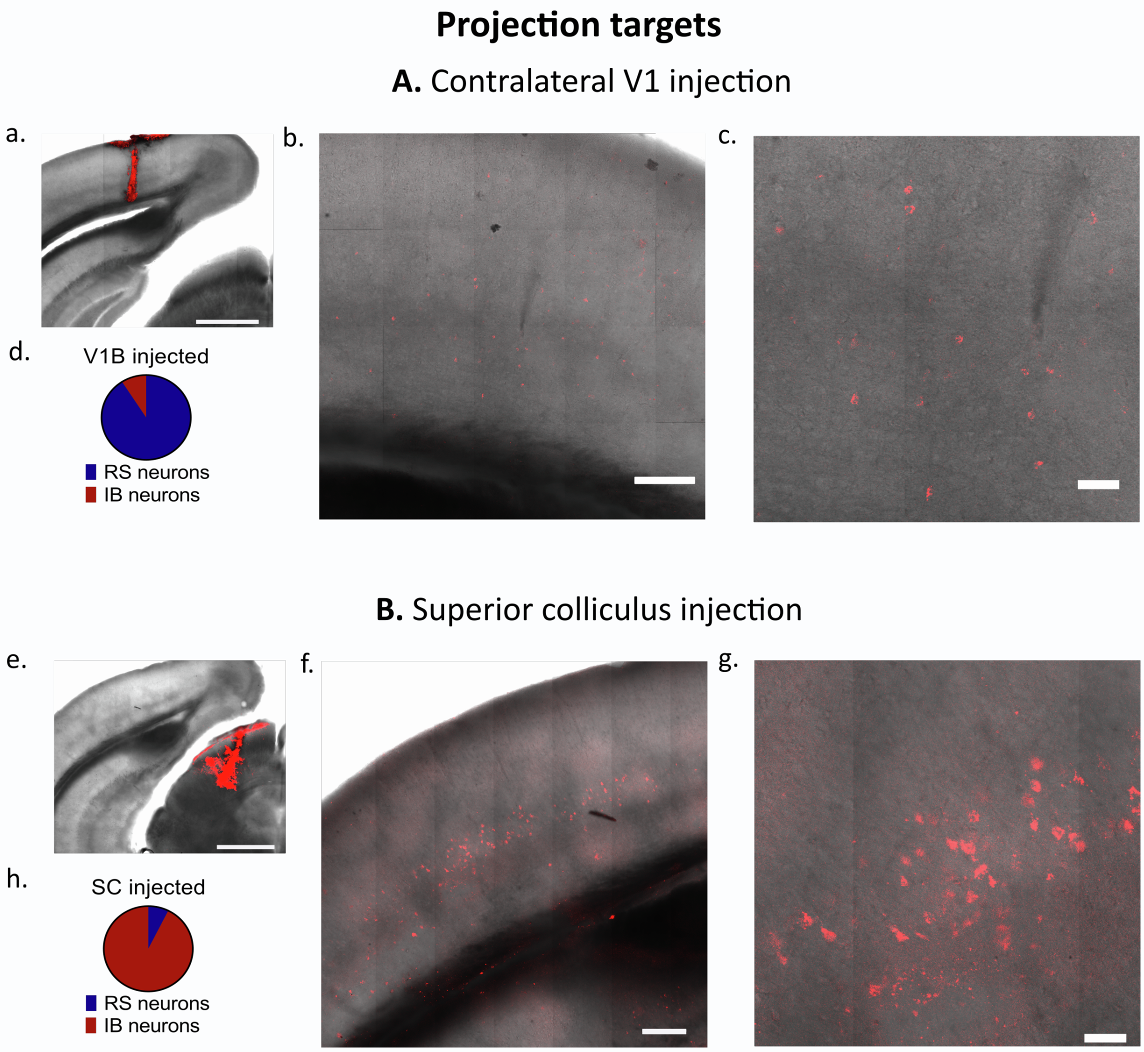

Figure S1. Coronal sections showing different projection targets of RS and IB neurons (related to Figure 1).

A. RS neurons project to contralateral visual cortex, while **B.** IB neurons project to superior colliculus (SC). **a.** Injection of retrobeads in V1b. Scale bar 1.00 mm **b.** Labelled neurons are found in all layers except layer I of visual cortex in the contralateral hemisphere. Scale bar 200μm. **c.** Magnified portion of a small area from panel b. Scale bar 50μm **d.** Pie chart representing proportion of RS and IB neurons among neurons projecting to contralateral visual cortex. **e.** Injection of retrobeads in superior colliculus. Scale bar 1.00 mm. **f.** Labelled neurons are mostly present in layer 5 of ipsilateral visual cortex. Scale bar 200μm. **g.** Magnified portion of a small area from panel f. Scale bar 50μm. **h.** Pie chart representing proportion of RS and IB neurons among neurons projecting to superior colliculus.

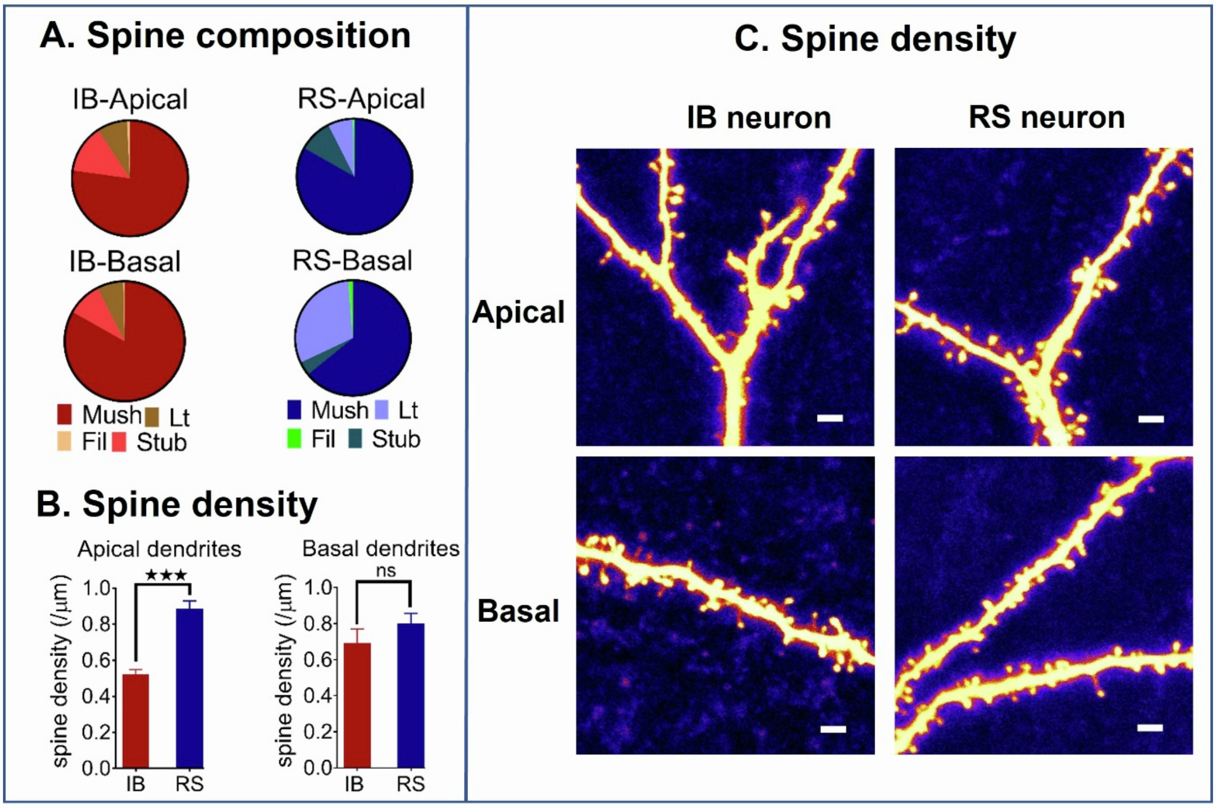

Figure S2. RS and IB neurons have different spine composition and density (related to Figure 1).

A. Pie charts showing basal dendrites of RS neurons have more long thin spines than the basal dendrites of IB neurons, while basal dendrites of IB neurons have more mushroom spines than basal dendrites of RS neurons (Mush – Mushroom spines, Lt- Long thin spines, Fil- Filopodia, Stub- Stubby spines). **B.** Apical dendrites of RS neurons have higher spine density than apical dendrites of IB neurons ($t_{(19)}$=7.26, $p<0.001$) while there is no difference in the spine density of basal dendrites of RS and IB cells. **C.** Representative images showing the above observations, scale bars – 2 µm.

*** $p<0.001$, ns not significant α=0.05. Histograms show means ± SEM.

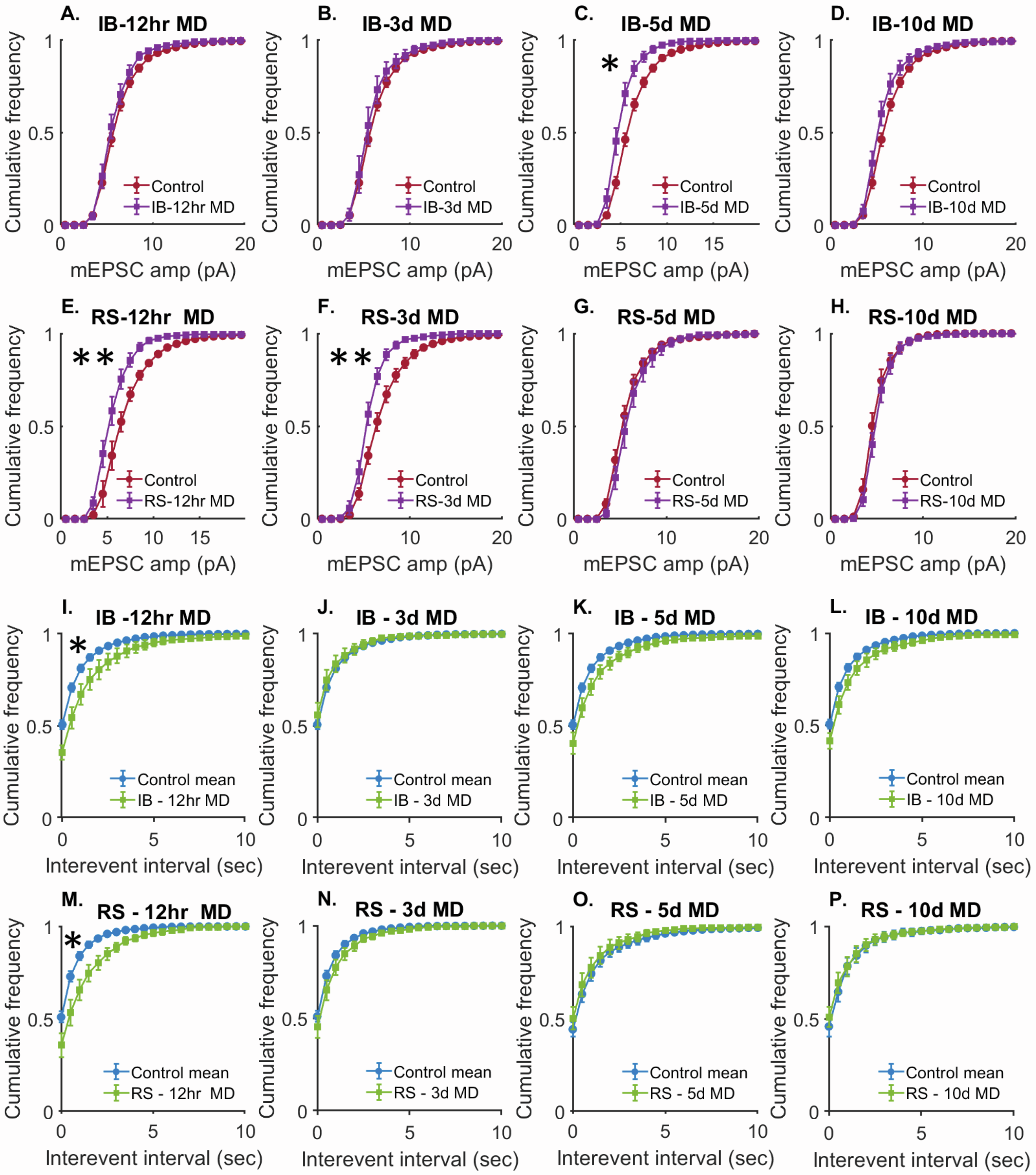

Figure S3. Effect of monocular deprivation on mEPSC amplitudes and inter-event intervals (related to Figure 3).

A-H: mEPSC amplitudes plotted in cumulative distribution functions (cdf). Control purple and MD maroon for IB (A-D) and RS cells (E-H). **A-D:** mEPSC amplitudes of IB neurons show reduction in mEPSC amplitudes after 5d MD, recovering to baseline after 10d of MD. **E-H:** mEPSC amplitudes of RS neurons show reduction after 12hr and 3d MD recovering to control on 5d MD. Bin interval-1pA.

I-L: mEPSC inter-event intervals plotted in cumulative distribution functions (cdf). Control blue and MD green for IB (I-L) and RS cells (M-P). **I-L**: Inter-event intervals increase for IB cells after 12 hours and recover after 3 days. **M-P:** RS neurons also show increase in mEPSC inter-event interval after 12 hours, followed by recovery at 3 days. Bin interval-0.5sec. *p<0.05, ** p<0.01. Plotted points are means ± SEM.

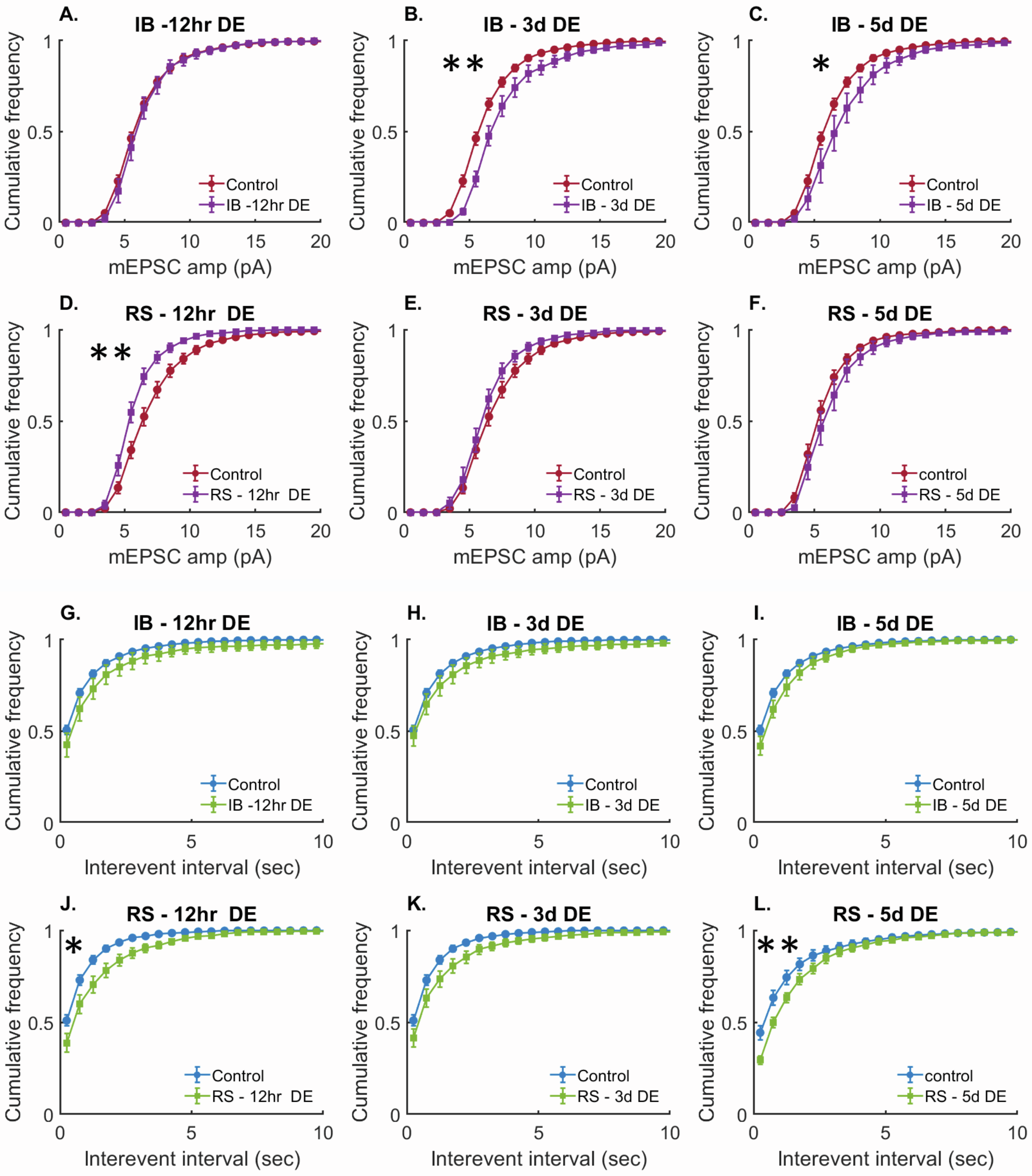

Figure S4. Effect of dark-exposure on mEPSC amplitudes and inter-event intervals (related to Figure 4).

A-F: mEPSC amplitudes plotted in cumulative distribution functions (cdf). Control purple and DE maroon for IB (A-C) and RS cells (D-F). **A-C:** mEPSC amplitudes of IB neurons show increases in mEPSC amplitudes after 3d and 5d DE. **D-F:** mEPSC amplitudes of RS neurons show reduction in mEPSC amplitudes after 12hr of DE recovering to control values by 3d DE. Bin interval-1pA.

G-L: mEPSC inter-event intervals plotted in cumulative distribution functions (cdf). Control blue and DE green for IB (G-I) and RS cells (J-L). **G-I: -** inter-event intervals of mEPSCs in IB neurons do not show any significant change with DE. **J-L: -** inter-event intervals appear to increase at all the time points for RS cells but the difference is significant only at 12 hours and 5d DE. Bin interval-0.5sec. $*p<0.05$, $** p<0.01$. Plotted points are means $\pm$ SEM.

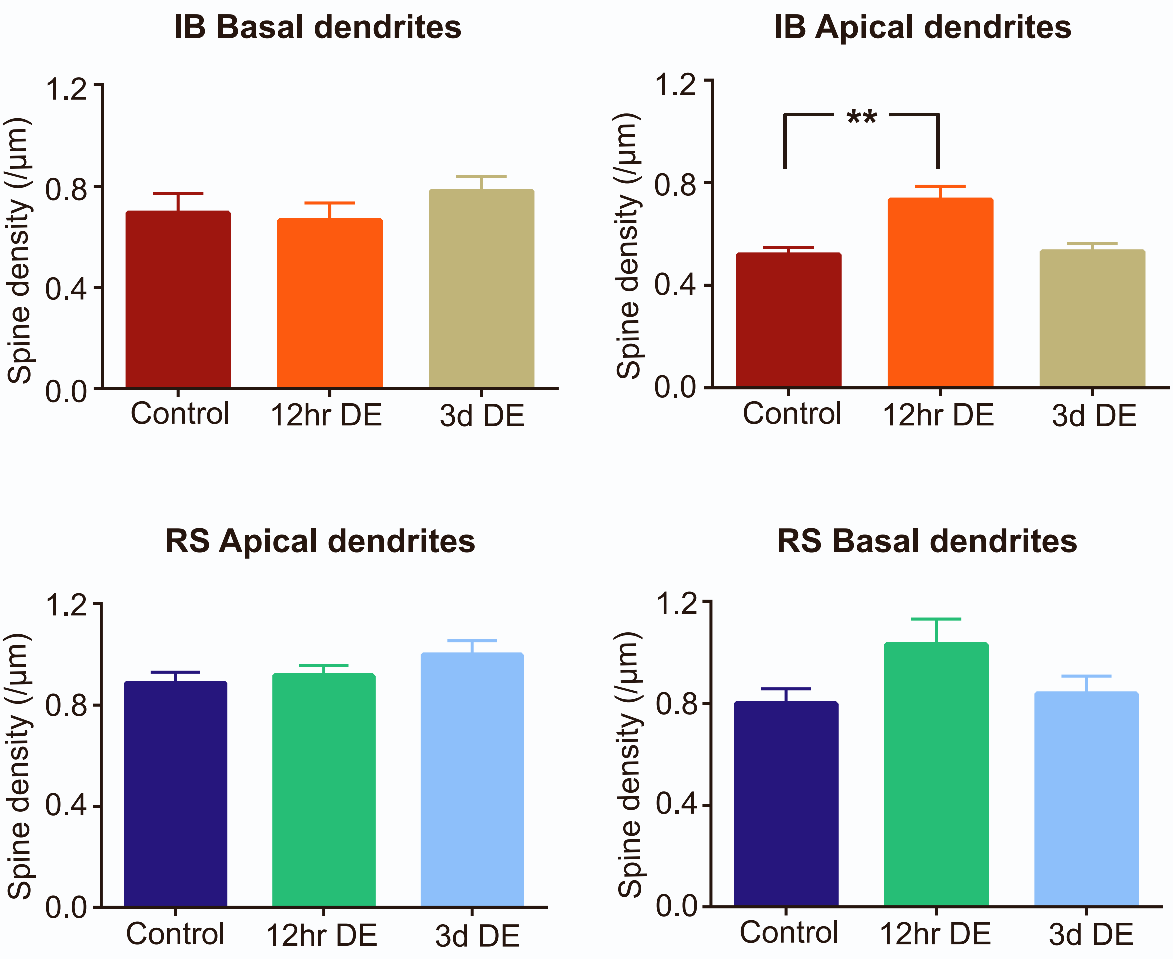

Figure S5. Effect of dark exposure (DE) on spine density (related to Figure 6).

A. DE does not change spine density on basal dendrites of IB neurons. **B.** On IB neurons' apical dendrites spine density increases with 12hr DE and comes back to baseline at 3d DE. In RS neurons DE has no significant impact on apical dendrites (**C**), or basal dendrites (**D**). ** $p<0.01$. Histograms represent means ± SEM.

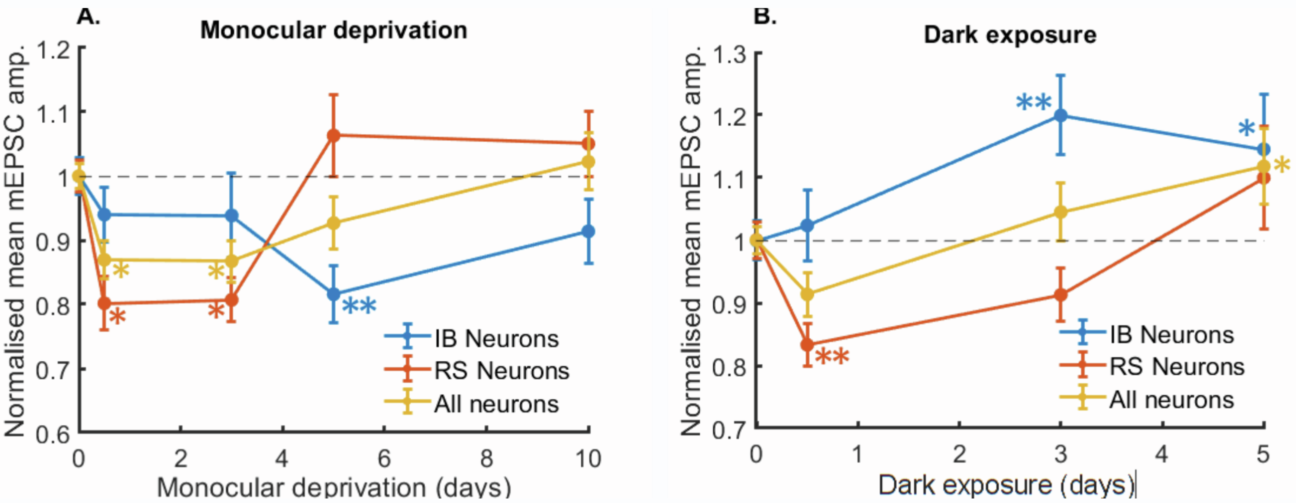

Figure S6. IB and RS data pooled together for MD and DE (related to Figures 3 and 4).

A: Layer 5 pyramidal neurons show Monocular Deprivation (MD) induced synaptic depression and a slow recovery toward baseline if data from IB and RS neurons are not treated separately (All neurons, yellow line and symbols). Note that IB (blue line and symbols) and RS neurons' responses (red line and symbols) exhibit different responses to MD, both from each other and from the pooled data.

B. Dark Exposure (DE) leads to small insignificant synaptic depression followed by late potentiation if the IB and RS neurons are not treated separately. The opposite direction of changes for RS and IB neurones largely cancel one another out. RS neurons (red line and symbols) only show depression and recovery, while IB neurons (blue line and symbols) only show potentiation due to DE. *$p<0.05$, ** $p<0.01$. Plotted points are means ± SEM.

AGE (days)	IB average IEI (s)	IB cell count	RS average IEI (s)	RS cell Count
26		0	1.207	2
27	0.151	1	0.925	6
28	1.248	8	0.738	11
29	0.647	7	0.573	3
30	0.850	2	0.913	5
31	0.797	3	1.039	1
32	0.952	5	1.349	3
33	1.190	11	1.217	10
34	0.946	10	1.558	10
35	0.623	2	1.516	6
36	0.739	3	0.794	5
38	0.729	1		0
TOTAL		**53**		**62**

Table S1. Analysis of changes in mEPSC inter-event interval as a function of age (related to Figure 2).

The median inter-event intervals for each cell are averaged for each age group and given in seconds. We found little correlation between age and inter-event interval over this period of development for Layer 5 RS (R^2 =0.18) and IB cells (R^2 = 0.01) and an ANOVA showed no significant effects (RS cells $F_{(1,61)}$=3.5, p=0.068; IB cells $F_{(1,52)}$=0.02, p=0.88).

Group	IB Animals	IB Neurones	RS Animals	RS Neurones	Age range (Days)
Young undeprived	19	27	16	31	27-32
Old undeprived	21	27	16	33	33-38
IB undeprived (all data)	40	54	--	--	27-38
MD 12 hour	7	9	7	9	27-30
MD 3 day	5	8	10	13	27-30
Control for MD 5 day	-	-	15	23	32-34 (RS)
MD 5 day	10	13	10	14	33-34 (RS), 31-34 (IB)
Control for MD 10 day	--	--	10	14	35-38 (RS)
MD 10 day	10	13	10	14	35-40 (RS), 34-40 (IB)
DE 12 hour	9	11	10	15	27-31 (RS), 27-31 (IB)
DE 3 day	9	12	12	14	28-31 (RS), 28-31 (IB)
DE 5 day	7	10	8	15	33-34 (RS), 33-34 (IB)
XPro control (12hr & 3d DE)	9	12	12	17	29-33 (RS), 28-33 (IB)
XPro 12 hour DE	8	13	9	13	28-34 (RS), 28-34 (IB)
XPro 3 day DE	7	8	7	13	29-32 (RS), 29-32 (IB)
XPro control (for 5 day DE)	6	8	6	10	31-33 (RS), 31-33 (IB)
XPro 5 day DE	6	8	8	11	31-33 (RS), 31-33 (IB)
T286 control	11	18	10	16	28-34 (RS), 28-34 (IB)
T286 12 hour DE	7	11	8	13	28-32 (RS), 28-31 (IB)
T286 3 day DE	6	12	5	9	30-33 (RS), 30-33 (IB)
T286 5 day DE	6	10	7	11	32-34 (RS), 32-34 (IB)
TOTALS	203	284	196	308	

Table S2. Number of animals and neurones in each subgroup (related to Star Methods). Note that MD = monocular deprivation, DE = dark exposure, XPro = treatment with XPro1595 and T286 = αCaMKII^{T286A} point mutant mice.
