# Supplementary material for: Differentiation of Hebbian and homeostatic plasticity mechanisms within layer 5 visual cortex neurons
Source: Cell Rep. 2022 May 31;39(9):110892. doi: 10.1016/j.celrep.2022.110892 (PMC9637998; doi:10.1016/j.celrep.2022.110892)
Supplement: Document S2. Article plus supplemental information [file mmc2.pdf]

Cell Reports

Article

Differentiation of Hebbian and homeostatic plasticity mechanisms within layer 5 visual cortex neurons

Graphical abstract

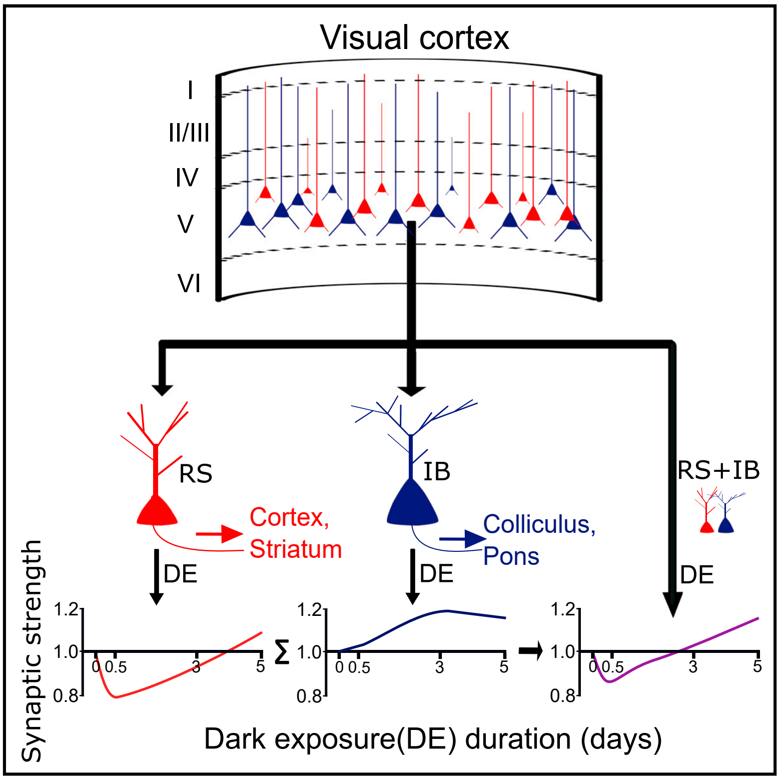

Highlights

- Layer 5 RS cell synapses mature during critical period for ocular dominance plasticity
- Layer 5 RS but not IB cells show TNF-α-dependent homeostatic recovery from depression
- Layer 5 IB but not RS cells show CaMKII-autophosphorylation-dependent potentiation
- Increases in spine head size precede functional potentiation in IB cells

Authors

Anurag Pandey, Neil Hardingham, Kevin Fox

Correspondence

foxkd@cardiff.ac.uk

In brief

Pandey et al. show that cells in the visual cortex projecting to different neuronal targets exhibit different synaptic plasticity mechanisms. While regular spiking (RS) cells show synaptic depression and exhibit TNF-α-dependent homeostatic recovery from depression, intrinsic bursting (IB) cells show potentiation that depends on CaMKII-autophosphorylation and not TNF-α.

Pandey et al., 2022, Cell Reports *39*, 110892
May 31, 2022 © 2022 The Authors.
https://doi.org/10.1016/j.celrep.2022.110892

CellPress

Cell Reports

CellPress
OPEN ACCESS

Article

Differentiation of Hebbian and homeostatic plasticity mechanisms within layer 5 visual cortex neurons

Anurag Pandey,[1] Neil Hardingham,[1] and Kevin Fox[1,2,*]
[1]School of Biosciences, Cardiff University Museum Avenue, Cardiff CF10 3AX, UK
[2]Lead contact
*Correspondence: foxkd@cardiff.ac.uk
https://doi.org/10.1016/j.celrep.2022.110892

SUMMARY

Cortical layer 5 contains two major types of projection neuron known as IB (intrinsic bursting) cells that project sub-cortically and RS (regular spiking) cells that project between cortical areas. This study describes the plasticity properties of RS and IB cells in the mouse visual cortex during the critical period for ocular dominance plasticity. We find that RS neurons exhibit synaptic depression in response to both dark exposure (DE) and monocular deprivation (MD), and their homeostatic recovery from depression is dependent on TNF-α. In contrast, IB cells demonstrate opposite responses to DE and MD, potentiating to DE and depressing to MD. IB cells' potentiation depends on CaMKII-autophosphorylation and not TNF-α. IB cells show mature synaptic properties at the start of the critical period while RS cells mature during the critical period. Together with observations in somatosensory cortex, these results suggest that differences in RS and IB plasticity mechanisms are a general cortical property.

INTRODUCTION

The cerebral cortex contains a diversity of excitatory neuronal subtypes. During development, a small set of relatively homogeneous progenitors give rise to several different types of adult neuron, expressing different molecules, displaying different dendritic morphologies, connecting to different neuronal circuits and projecting to different targets in the brain (Lake et al., 2016; Lodato and Arlotta, 2015). Among the projection cells, layer 5 neurons can be classified broadly into those that project sub-cortically, to targets such as the superior colliculus and pontine nuclei and those that project cortico-cortically between different cortical areas. The projections of these neurons correlate with their intrinsic membrane properties, their neuronal morphology and their intracortical connectivity (Hattox and Nelson, 2007; Kasper et al., 1994; Schubert et al., 2006). The sub-cortically projecting neurons produce bursts of action potentials when depolarized (intrinsic bursting [IB] cells), have highly branched apical dendrites, and receive inputs from several cortical columns, whereas the cortico-cortically projecting neurons tend to fire regular trains of action potentials when depolarized (regular spiking [RS] cells) (Agmon and Connors, 1992; Hattox and Nelson, 2007), have little or no branching of the apical dendrite, and mainly receive inputs from within the cortical column (Hattox and Nelson, 2007; Schubert et al., 2007). In addition, RS cells either project to the striatum and cortex or just cortico-cortically (Kim et al., 2015). Recently, studies in the barrel cortex have shown that the differences exhibited by layer 5 RS and IB cells also extend to their plasticity mechanisms (Greenhill et al., 2015). While IB cells show αCaMKII-dependent potentiation and little depression during patterned whisker deprivation, RS cells show strong experience-dependent depression and little αCaMKII-dependent potentiation, instead exhibiting TNF-α-dependent homeostatic plasticity, which is characteristic of synaptic scaling (Greenhill et al., 2015; Jacob et al., 2017).

These studies raise the question of whether the divergent plasticity mechanisms seen in layer 5 neurons are a specialized feature of the barrel cortex or a broader property of cortical organization. To test this idea, we needed to study plasticity in RS and IB cells in other cortical areas. Given that the visual cortex is highly plastic and that experience-dependent plasticity has been studied extensively in this structure (Hooks and Chen, 2020; Wiesel and Hubel, 1963), we sought to determine whether layer 5 neurons showed different plasticity mechanisms in the IB and RS cells of the visual cortex.

Plasticity in the visual cortex is thought to operate partly via synaptic scaling (Kaneko et al., 2008; Turrigiano et al., 1998), a process originally studied by application of TTX to neuronal cultures *in vitro* (Stellwagen and Malenka, 2006; Turrigiano et al., 1998). Two conditions are known to lead to TNF-α-dependent up-scaling processes in the visual cortex; dark exposure (DE) *in vivo* causes up-scaling of synaptic weights (Ranson et al., 2012) and eye enucleation causes enlargement of the surviving dendritic spines on dendrites that have lost spines (Barnes et al., 2017). However, other studies have implicated NMDA receptors and CaMKII-autophosphorylation in plasticity induced by DE, both of which would suggest that an LTP-type mechanism is also involved (Cooke and Bear, 2010; Daw et al., 1999;

This is an open access article under the CC BY license (http://creativecommons.org/licenses/by/4.0/).

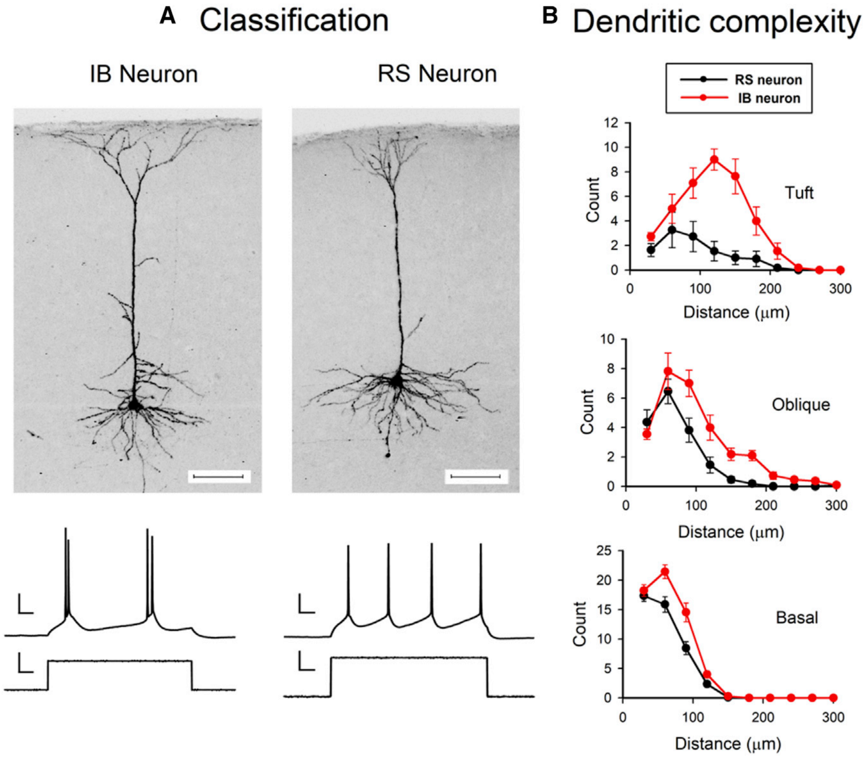

Figure 1. IB and RS neuronal properties

(A) Example morphology of electrophysiologically identified IB and RS cells. Scale bars, 100 μm. The spike discharges are produced from somatic current injection. Scale bars, 20 mV and 100 ms (spike discharges) and 20 pA and 100 ms (current injection).

(B) Sholl plots for apical tuft, oblique, and basal dendrites from RS (black) and IB cells (red). RS neurons have significantly fewer branches than IB cells at apical tuft ($t_{(20)} = 5.03$, $p < 0.0001$) apical oblique ($t_{(20)} = 2.87$, $p < 0.01$), and basal dendritic locations ($t_{(20)} = 3.13$, $p < 0.01$).

Data points represent means ± SEM. See also Figures S1 and S2 for further distinctions between RS and IB cells.

Gu et al., 1989; Kirkwood et al., 1997). Thus far, both Hebbian and synaptic scaling mechanisms have mainly been studied in layer 2/3, so an additional question arises about which, if either, apply to layer 5.

We found that the mechanisms employed by neurons for TNF-α-dependent homeostatic responses are specific to cortical projection subtypes. RS cells projecting cortico-cortically, do indeed exhibit homeostatic up-scaling dependent on TNF-α processing following experience-dependent synaptic depression; however, IB cells projecting sub-cortically do not; instead, they show a response that depends on αCaMKII-autophosphorylation, a key molecular process required for LTP. The subtype divergence of plasticity mechanism is therefore not a peculiarity of the barrel cortex but extends to the visual cortex, strongly suggesting that plasticity subtypes are a cortex-wide phenomenon.

RESULTS

IB and RS neuron characteristics

IB and RS neurons were characterized electrophysiologically by recording their firing patterns in response to somatic current injection (Figure 1A). We found that the electrophysiological classification correlated well with several morphological characteristics. Sholl analysis revealed that dendrites were generally more branched in IB than in RS neurons ($F_{(1,1)} = 41.24$, $p < 0.0001$) and that this was true for basal, apical tuft, and apical oblique dendrites (Figure 1B). In addition, the span of the apical tuft in layer I was consistently far broader in IB cells (average = 280 ± 24 μm) than in RS cells (average = 212 ± 30 μm) and significantly different ($t_{(16)} = 1.78$, $p < 0.05$), which resulted in IB cells' apical dendrites spanning a greater horizontal distance than their basal dendrites (80% of cases) while RS cells' apical tufts spanned the same (within 10%) or a smaller radius than the basal dendrites (100% of cases). We also found that the electrophysiological identity of the neurons correlated with their projection targets (Figure S1). Neurons projecting to the superior colliculus showed a burst of spikes in response to somatic current injection (93%). Similarly, neurons that projected to the opposite hemisphere of the visual cortex had a 91% chance of exhibiting an RS response to somatic current injection (Figure S1). These characteristics confirm that our electrophysiological categorization of IB and RS neurons was consistent with those of previous studies (Gao and Zheng, 2004; Hattox and Nelson, 2007; Kasper et al., 1994; Schubert et al., 2006).

We discovered one morphological feature concerning spine type that has not previously been reported to be different between RS and IB cells. The distribution of spine types was different in RS and IB cells (Figure S2). RS cells had a greater proportion of long thin spines (average 30% in RS cells versus 7% in IB), while IB cells had a greater proportion of mushroom-shaped spines (average 64% in RS cells versus 83% in IB). These two spine types made up the majority of the total for both cell types, with stubby and filopodial spine types constituting only 6%–10%. The difference in the proportion of spine types between RS and IB cells was highly statistically significant ($\chi^2 = 23$, $df = 2$, $p < 0.001$).

We also found a difference in spine density in IB versus RS cells. A two-way ANOVA showed an effect of cell type on spine density ($F_{(1,1)} = 18.12$, $p < 0.0002$) and an interaction between the location of the spines (apical versus basal) and the cell type (IB versus RS) ($F_{(1,1)} = 5.46$, $p < 0.03$). In general, IB cells had a lower spine density (by 28%) than RS cells (Table 1), but this was most noticeable on the IB cells' apical dendrites, where the spine density (of 0.52 spines per μm) was approximately 40% lower than on IB cell basal dendrites or any location on RS cells (post-hoc t test, α = 0.05) (Figure S2). Taken together with the spine category differences, these observations suggest that RS cells have

Table 1. Average statistics for control conditions

	Input resistance (MΩ)	Spine density (spines per μm)	mEPSC amplitude (pA)	Age range (days)
RS young undeprived	201 ± 9, n = 34	0.84 ± 0.04 n = 11	7.51 ± 0.29, n = 31	27–32
RS old undeprived	215 ± 10, n = 34	N/A	5.85 ± 0.21, n = 33	33–38
IB undeprived	167 ± 7, n = 54	0.61 ± 0.04, n = 11	6.84 ± 0.20, n = 54	27–38

Input resistance, spine density, and mEPSC amplitudes are shown for the two RS cell age groups (young = 27–32 days; old = 33–38 days) and the IB cells (27–38 days).

a higher spine density comprising more thin spines than IB cells and conversely, IB cells have a lower spine density but a greater proportion of mushroom spines.

Development

During the critical period for ocular dominance plasticity (P19-32) (Gordon and Stryker, 1996), mEPSC amplitudes in layer 5 RS cells decreased with age, whereas those for IB cells remained constant with age (Figure 2; Table 1). We compared mEPSCs in two age groups, P27-32 and P33-38. A two-way ANOVA showed a strong effect of age on mEPSC amplitude ($F_{(1,1)}$ = 7.87, $p < 0.01$) and an interaction between age and cell type ($F_{(1,1)}$ = 12.43, $p < 0.001$). Post-hoc t tests showed that this was because, earlier in the critical period, the average mEPSC amplitude for RS cells was higher (at 7.5 pA) compared with at the end (5.8 pA), and the two values were highly significantly different ($t_{(62)}$ = 4.97, $p < 0.001$), whereas mEPSCs for IB cells were similar at the two ages (6.7 and 6.9 pA) and not statistically different ($t_{(53)}$ = 0.21, p = 0.65). In corroboration, we also found the linear regression fit for a plot of mEPSC amplitude versus age had a negative slope significantly different from zero for RS cells ($F_{(1,9)}$ = 13.2, $p < 0.01$) and correlated with the data (R^2 = 0.59), whereas the regression fit for IB cells was flat ($F_{(1,9)}$ = 0.71, p = 0.42) and uncorrelated with the data (R^2 = 0.07).

We tested to see whether other cell properties changed as a function of development. We found that neither RS nor IB neurons (Table 1) showed changes in input resistance over this period of development (RS: P27-32: R_{in} = 245 ± 16.8 MΩ; P33-38: R_{in} = 251 ± 18.5 MΩ; $t_{(51)}$ = 0.87, p = 0.38; IB: P27- 32: R_{in} = 163 ± 9.8 MΩ; P33-38: R_{in} = 171 ± 10.2 MΩ; $t_{(52)}$ = 0.62, p = 0.53). We also found that mEPSCs' frequency did not change significantly during the developmental time window studied (Table S1). Inter-event intervals of mEPSCs were not different between the P27 and P33-38 age groups, neither for RS cells (Wilcoxon test on median inter-event intervals, χ^2 = 2.32, df = 1, p = 0.12) nor for IB cells (χ^2 = 0.0, df = 1, p = 1.0).

We conclude that layer 5 RS cells continue to develop throughout the critical period, reducing their synaptic amplitude, while IB cells have either already completed this stage of development or do not develop this way at all. Therefore, in the results described below, we have compared the experimental data against closely age-matched undeprived controls for RS cells, while the IB cells are compared across the P27 to P38 time period.

Monocular deprivation

Monocular deprivation (MD) is the classic method for investigating plasticity in the visual cortex (Gordon and Stryker, 1996; Heynen et al., 2003; Wiesel and Hubel, 1963) and has been shown to induce Hebbian and homeostatic components of plasticity (Toyoizumi et al., 2014). However, studies have mainly been directed at layer 2/3 and layer 4 cells and, where studies have addressed plasticity in layer 5, they have usually not differentiated between RS and IB neurons (but see Medini, 2011b).

For RS neurons located contralateral to the closed eye and in the binocular zone (see STAR Methods), we found that MD caused a rapid depression in mEPSC amplitude after 12 h that was sustained to 3 days, but which showed a homeostatic rebound to baseline values at 5 days (Figure 3). An ANOVA showed a strong effect of deprivation on age-matched mEPSC amplitude ($F_{(4,4)}$ = 6.45, $p < 0.0001$) and post-hoc t tests showed that only the 12 h and 3 day time points were different from undeprived age-matched controls (12 h, $t_{(34)}$ = 2.87, $p < 0.01$; 3d, $t_{(38)}$ = 3.2, $p < 0.005$).

The IB neurons showed a delayed effect of deprivation in comparison with the RS neurons. The mEPSC amplitudes at the 5 day time point showed depression compared with controls (Figure 3). An ANOVA showed an effect of deprivation ($F_{(4,96)}$ = 2.5, $p < 0.05$), but none of the deprivation time points were distinguishable from controls apart from the 5 day time point ($t_{(65)}$ = 2.87, $p < 0.01$). A homeostatic rebound occurred between 5 and 10 days (Figure 3). The IB cells therefore behaved similarly to the RS cells but delayed both in depression onset and rebound.

We also tested to see whether the frequency of mEPSCs was affected by MD and found that MD had an effect on mEPSC frequency in both IB and RS cells, but only at the 12 h time point (Figure S3: I, M). Inter-event intervals of mEPSCs were increased at 12 h both for IB cells (Wilcoxon test on median inter-event intervals, χ^2 = 4.92, df = 1, $p < 0.05$) and RS cells compared with age-matched controls (χ^2 = 5.36, df = 1, $p < 0.05$). The reduction in mEPSC frequency at 12 h would be expected to exacerbate the effect of the reduced mEPSC amplitude for the RS cells.

In addition, we tested whether input resistance (R_{in}) changed during deprivation. We found that 12 h of MD increased input resistance in both RS and IB cells, but that it returned to control values for all other time points. A two-way ANOVA showed an effect of cell type ($F_{(1,1)}$ = 16.03, $p < 0.001$) and deprivation ($F_{(4,4)}$ = 2.76, $p < 0.05$) but no interaction. IB cells had lower input resistance than RS cells (IB cells 167 ± 7.5 MΩ versus RS cells 208 ± 6.7 MΩ) (Table 1), and the difference was highly significant ($t_{(210)}$ = 4.0, $p < 0.001$). Post-hoc t tests also showed that R_{in} increased only at the 12 h time point ($t_{(210)}$ = 3.3, $p < 0.002$) independent of cell type. The increase in R_{in} at 12 h may have some effect in offsetting the synaptic depression at 12 h in RS cells but, because it is not sustained beyond this time point, it would not affect the visual responses at 3 days of MD.

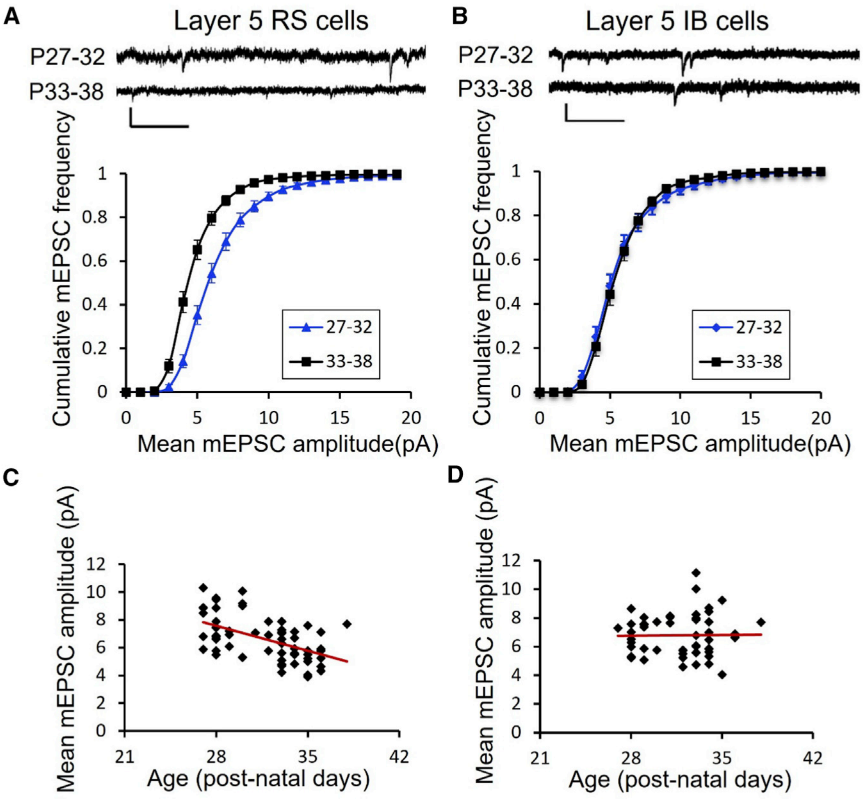

Figure 2. Synaptic development of layer 5 RS and IB neurons

(A) Example mEPSC traces and cumulative distribution functions (CDFs) for RS cells in the P27-32 (blue) and P33-38 (black) age group. Scale bars, 10 pA and 250 ms. The CDFs for the two age groups are significantly different ($p < 0.001$, see results). Data points represent means ± SEM.

(B) Example mEPSC traces and CDFs for IB cells in the P27-32 (blue) and P33-38 (black) age group. Scale bars, 10 pA and 250 ms. The CDFs for the two age groups are not significantly different ($p = 0.65$, see results).

(C and D) RS cells show a decrease in synaptic efficacy between P27 and P38 (linear regression, $p < 0.01$), whereas (D) IB cells show no change over this period (linear regression, $p = 0.42$; see text for statistics). Each data point represents a single cell.

Dark exposure

DE has been used as a model for inducing synaptic scaling in layer 2/3 visual cortical neurons (Ranson et al., 2012). In this study, we wanted to determine whether layer 5 RS and IB cells both underwent synaptic scaling and to what extent the process was homeostatic.

A two-way ANOVA on mEPSC amplitudes showed an effect of cell type ($F_{(1,1)} = 11.85$, $p < 0.001$), duration of DE ($F_{(3,3)} = 4.05$, $p < 0.01$), and an interaction between the two ($F_{(3,3)} = 3.95$, $p < 0.02$). Analyzing the effect of DE on RS cells separately, post-hoc t tests with age-matched controls showed that DE caused a rapid depression of mEPSC amplitudes after 12 h ($t_{(46)} = 2.76$, $p < 0.01$), which returned to baseline after 3 days ($t_{(45)} = 1.05$, $p = 0.29$), similar to the effect of MD on RS cells but with a faster homeostatic return to baseline (Figure 4).

The effect of DE on IB neurons was fundamentally different from its effect on RS cells in that it caused a clear potentiation, rather than a depression and homeostatic rebound (Figure 4). Post-hoc t tests showed that potentiation was significant at both the 3 day ($t_{(65)} = 2.85$, $p < 0.01$) and 5 day time points ($t_{(63)} = 2.44$, $p < 0.02$).

We also tested whether DE altered the inter-event intervals of mEPSCs (Figure S4) and found that it did not in IB cells at any time point (KW test on median inter-event intervals, $\chi^2 = 3.0$, df = 3, $p = 0.39$). However, inter-event intervals were increased for RS cells specifically at the 12 h (Wilcoxon test, $\chi^2 = 4.2$, df = 1, $p < 0.05$) and the 5 day time points ($\chi^2 = 6.7$, df = 1, $p < 0.01$) compared with age-matched controls. The reduction in mEPSC frequency might therefore exacerbate the effect of the reduction in mEPSC amplitude at 12 h and to some extent offset the recovery of the mEPSC amplitude to baseline values at 5 days (Figure 4A).

TNF-α and αCaMKII-autophosphorylation dependence of plasticity

The different responses of RS and IB cells to DE suggest that different plasticity mechanisms may underlie the effects. The RS cells show depression followed by homeostatic rebound to control values, which is a signature of homeostatic plasticity previously found to depend on TNF-α (Greenhill et al., 2015; Ranson et al., 2012). We tested to see whether plasticity was TNF-α dependent using the injectable TNF-α dominant-negative peptide XPro1595. XPro1595 was administered by i.p. injection 12 h before the DE period began (see STAR Methods). XPro1595 combines with endogenous extracellular TNF-α and renders the resultant trimeric agonist ineffective at binding at TNF-α receptors (Steed et al., 2003).

We found that XPro1595 had no effect on the depression induced in RS cells by DE, but that it prevented the homeostatic rebound in the mEPSC amplitude that occurred between 3 and 5 days in untreated animals (Figure 5). A two-way ANOVA showed an effect of DE and XPro1595 on the mEPSC amplitude and an interaction between the two ($F_{(3,3)} = 5.7$, $p < 0.005$). The interaction arose from the 5 day time points being different between XPro1595-treated and untreated cases ($t_{(24)} = 3.46$, $p < 0.001$), while none of the other time points were different (t test, $\alpha = 0.05$). In contrast to the effect on RS cells, XPro1595 had no effect on IB cells (Figure 5D). These results suggest that the rebound observed in RS cells toward baseline response levels is TNF-α-dependent, while the potentiation seen in IB cells is not TNF-α dependent.

Previous studies in the barrel cortex had shown potentiation in layer 5 IB cells to be dependent on αCaMKII-autophosphorylation (Greenhill et al., 2015). CaMKII-t286a mice are point

CellPress
OPEN ACCESS

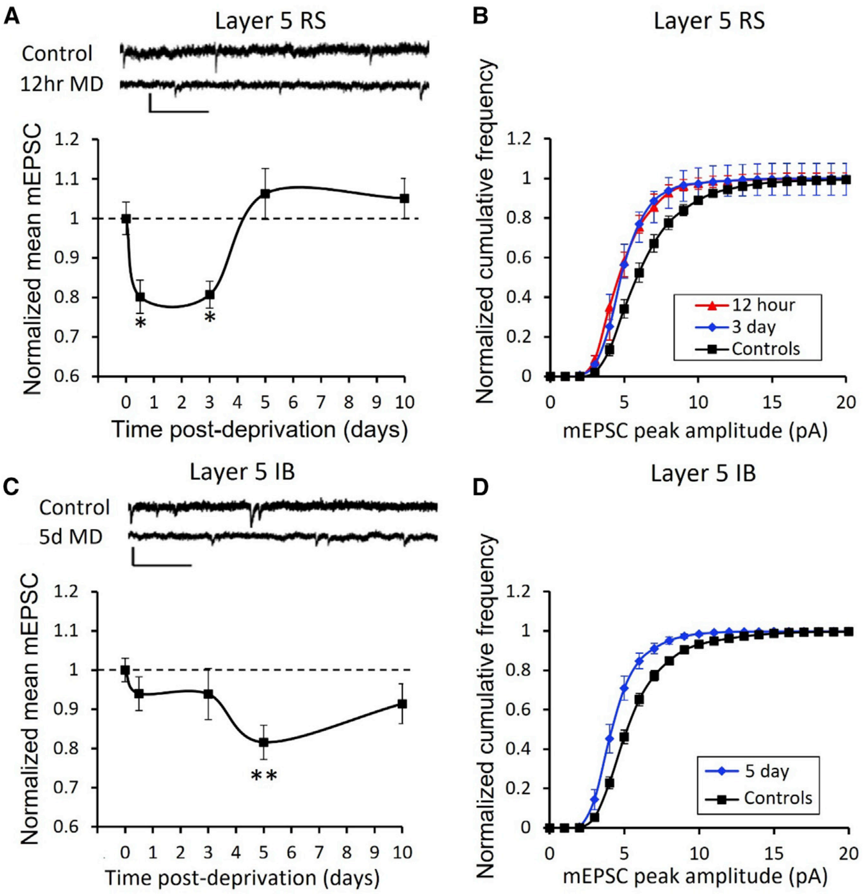

Figure 3. Effect of monocular deprivation on synaptic strength in layer 5 RS and IB cells

(A) Example mEPSCs and time course of change in RS cells' mEPSCs following monocular deprivation. Scale bars, 10 pA and 250 ms.

(B) CDFs for control and the two significantly depressed time points shown in (A) at 12 h (red) and 3 days (blue).

(C) Example mEPSC traces and time course of changes in IB cells' mEPSCs following monocular deprivation. Scale bars, 10 pA and 250 ms.

(D) CDFs for the 5-day time point (blue) compared with control (black). *p < 0.05, **p < 0.01. Data points represent means ± SEM. (See Figure S3 for CDFs of all time points). See also Figure S6.

mutants that have an alanine substituted for the threonine at the autophosphorylation site (Giese et al., 1998). The mice have calcium-sensitive CaMKII but lack the ability to autophosphorylate at the threonine 286 site. The autophosphorylation step is necessary for LTP to be sustained *in vitro* (Chang et al., 2017; Giese et al., 1998; Hardingham et al., 2003) and is also important for dendritic spine enlargement *in vivo* (Seaton et al., 2020). We therefore tested whether the potentiation of the mEPSCs produced in IB cells was CaMKII-autophosphorylation dependent.

We found that potentiation produced by 3–5 days DE was absent in layer 5 IB cells in mice carrying the mutation preventing CaMKII-autophosphorylation (CaMKII-t286a mice), as shown in Figure 5C. A two-way ANOVA showed an effect of genotype ($F_{(3,3)} = 4.7$, $p < 0.005$) and post-hoc t tests showed that this was because the 3 and 5 day time points were different in wild-type mice compared with the CaMKII-t286a point mutants (3 day $t_{(22)} = 2.27$, $p < 0.05$, 5 day $t_{(18)} = 1.96$, $p < 0.05$). In contrast, the depression and homeostatic response of the RS cells recorded in these mutants was the same as found in the wild-type animals. A two-way ANOVA showed no difference between RS cells' response to DE in wild-type and mutant cells, only an effect of deprivation ($F_{(3,3)} = 5.09$, $p < 0.005$). These findings therefore show that the potentiation seen in IB cells depends on the same molecular mechanism as LTP, experience-dependent potentiation and experience-dependent dendritic spine enlargement, whereas the depression and rebound homeostatic potentiation seen in the RS neurons utilizes different mechanisms.

Morphological correlates of changes in mEPSCs

We looked at the morphology of dendritic spines to see if we could identify structural changes that corresponded to the electrophysiological changes we observed with DE. Consistent with other studies, we found that spine head sizes were log-normally distributed (Loewenstein et al., 2011). In IB cells, we found that there was an effect of DE on spine head size and that, in the undeprived case, was significantly different between apical and basal dendrites. A two-way ANOVA showed an effect of dendritic location ($F_{(1,1)} = 12.6$, $p < 0.001$) and deprivation on spine head size ($F_{(2,2)} = 5.7$, $p < 0.01$). Post-hoc t tests showed that spine head sizes were far smaller on basal dendrites when compared with apical dendrites ($t_{(21)} = 12.0$, $p < 0.003$). Spine head size increased after just 12 h of deprivation on basal dendrites (compared with control underived cases, $t_{(20)} = 15.8$, $p < 0.0001$) before regressing back toward control values after 3 days (Figure 6A). What appear to be changes in apical dendritic spine size with deprivation were not statistically significant ($t_{(20)} = 2.4$, $p = 0.13$). An ANOVA did not reveal any differences in spine head size in RS neurons with DE; although it appeared that apical dendrites may have enlarged at 12 h (Figure 6B), this was not statistically significant ($t_{(18)} = 2.1$, $p = 0.16$).

We found that apical dendritic spine sizes were only different from basal dendritic spines in undeprived cases (Figure 6A) and that DE negated the difference. The increase in spine head size after 12 h appeared to be due to a small (60 nm) and relatively uniform rightward shift in spine size values (Figure 6C). The difference in spine sizes between apical and basal dendrites (Figure 6D) is likely to be related to distance-dependent scaling (Magee and Cook, 2000), which is

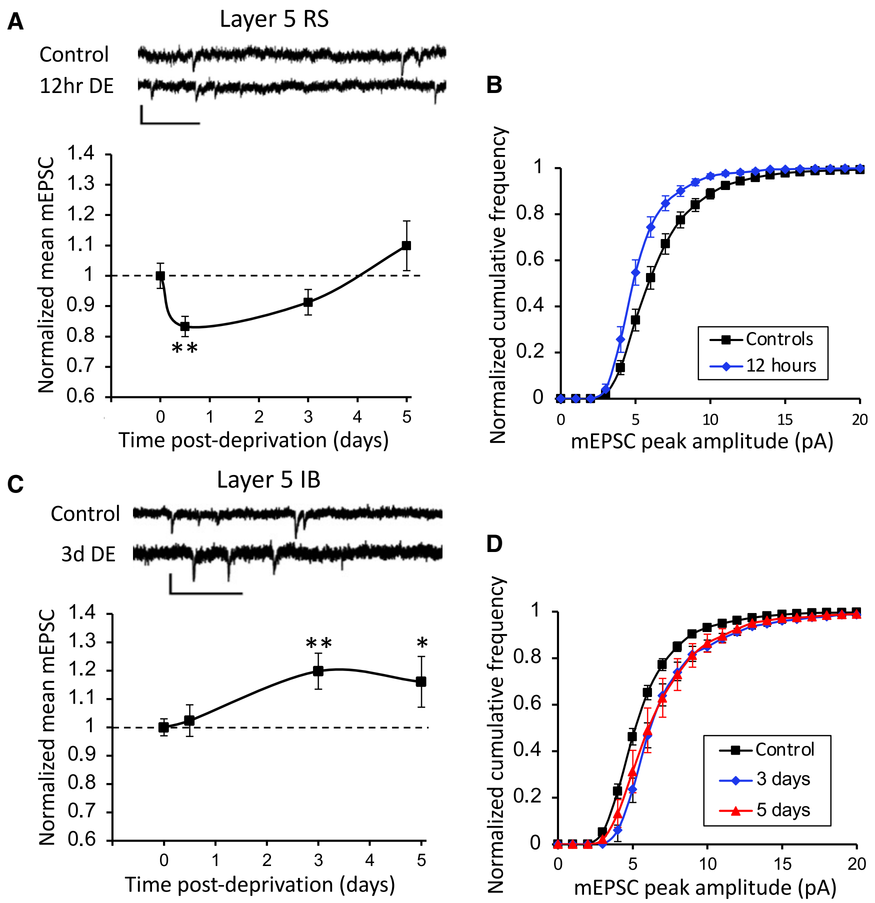

Figure 4. Effect of dark exposure on synaptic strength in layer 5 RS and IB cells

(A) Example mEPSC traces and time course of change in RS cells' mEPSCs following dark exposure. Scale bars, 10 pA and 250 ms.

(B) CDFs for control (black) and the 12 h DE (blue) significantly depressed time point shown in (A). Scale bars, 10 pA and 250 ms.

(C) Example mEPSC traces and time course of changes in IB cells' mEPSCs following dark exposure.

(D) CDFs for the 3-day DE (blue) and 5-day DE (red) time points. $^{*}p < 0.05$, $^{**}p < 0.01$. Data points represent means ± SEM. (See Figure S4 for all CDFs). See also Figure S6.

thought to compensate for dendritic EPSP attenuation distant from the soma.

The changes in spine head size occurred in the absence of any overall change in spine density on the basal dendrites of RS or IB cells (Figure S5). However, we did observe an increase in the spine density on apical dendrites of IB cells (but not RS cells) after 12 h of deprivation (two-way ANOVA; interaction between spine location and deprivation, $F_{(2,2)} = 4.3$, $p < 0.02$).

Our structural plasticity findings therefore show that, for IB cells, changes in basal spine head size and apical spine density occur after 12 h of deprivation, before any electrophysiological changes are detectable in the mEPSC amplitudes (compare Figures 4C and 6A). Conversely, when the electrophysiological changes are seen in IB cells at 3 days, the structural changes have decreased slightly. For RS cells, we did not find any overt structural changes in spine density or spine head size that might correlate with the reduction in mEPSC amplitudes seen after 12 h of deprivation.

DISCUSSION

Generality of RS/IB dichotomy

One of our objectives was to test whether the distinction between IB and RS synaptic plasticity properties can be generalized across cortical areas. Despite the very different nature of visual and somatosensory information, we found that IB and RS cells exhibited the same differences in plasticity mechanisms in visual cortex as they did in the somatosensory cortex. In both cortical areas, IB cells showed potentiation beyond baseline values: in the visual cortex in response to DE (Figure 4C) and in the barrel cortex in response to chessboard pattern whisker deprivation (Greenhill et al., 2015). The IB cell potentiation was also dependent on CaMKII-autophosphorylation in both areas and not dependent on TNF-α (in either cortical area). Similarly, RS cells in both cortical areas showed synaptic depression in response to sensory deprivation, independent of the type of deprivation; MD or DE for visual cortex (Figures 3A and 4A) and chessboard pattern deprivation or all -whisker deprivation in the barrel cortex (Greenhill et al., 2015). In both cases, the homeostatic rebound in the response of the RS cells followed a similar time course in the two cortical areas (of approximately 3 days) and was dependent on TNF-α and not CaMKII-autophosphorylation.

Interpreting visual cortex plasticity experiments

Our studies show that the time course and direction of plasticity are different in RS and IB cells, which needs to be considered when understanding the effect of MD and/or DE. In fact, a different interpretation emerges if we ignore the distinction between cell types. Figure S6 shows a plot of the time course of synaptic strength during DE and MD disregarding cell type. Under these circumstances, the mean EPSC amplitudes appear to show a small depression and return to baseline following either form of deprivation; for DE, the RS and IB cell EPSC amplitudes move in opposite directions almost canceling one another out so that the only time point that differs from the aggregated control data is the 5 day time point, which shows a small potentiation (of 12%, $t_{(128)} = 5.8$, $p < 0.02$). For MD, the combined RS and IB populations show depression (maximum at 3 days, 14% reduction, $t_{(124)} = 8.6$, $p < 0.005$), as was observed with the individual

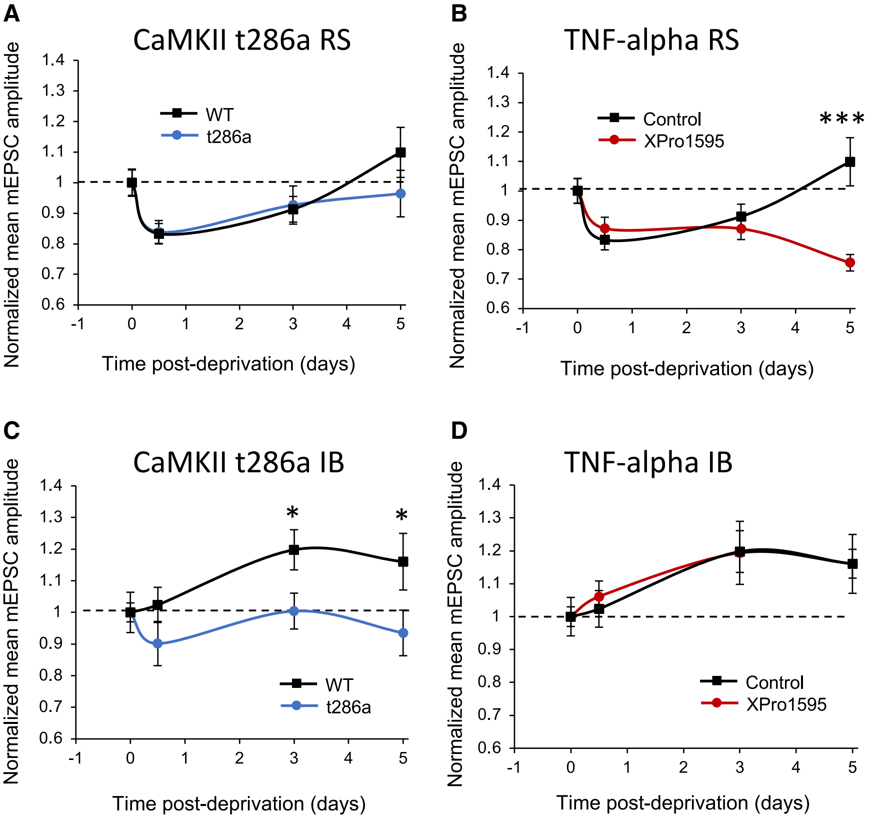

Figure 5. RS and IB neurons show different plasticity mechanisms during dark exposure

(A and B) Depression and homeostatic rebound to baseline values in wild-type cells (black) and CaMKII t286a mutants (blue) are similar in RS cells (A), whereas homeostatic rebound does not occur in wild-type RS cells treated with TNF-α inhibitor (red) (B).

(C and D) Potentiation does not occur in IB cells of CaMKII-t286a mutants (blue) (C); however, potentiation is unaffected by treatment with TNF-α inhibitor (red) (D). Data points represent means ± SEM. (* indicates $p < 0.05$ and ***$p < 0.001$)

cell types, but again the effect of the RS and IB cells moving in opposite directions between 3 and 5 days cancel one another out such that the homeostatic rebound is only complete after 10 days, whereas for RS cells this actually takes just 5 days.

The similarity of the RS cells' reaction to MD and DE can probably be explained most easily by their strong contralateral bias (Medini, 2011a), since we recorded neurons in the hemisphere contralateral to the MD. The RS cells were therefore deprived of their dominant contralateral input during MD, while the ipsilateral input was additionally deprived during DE, which would not have much additional effect on the RS cells as they are strongly contralateral biased.

Cellular diversity in plasticity mechanisms

While the RS cells show a classic depression followed by TNF-α-dependent synaptic scaling, IB cells show a mechanism more closely related to LTP, judging by its dependence on CaMKII-autophosphorylation. Recent studies regarding the effects of DE on layer 2/3 cells in visual cortex have shown that plasticity occurs via NMDA receptors rather than synaptic scaling (Bridi et al., 2018). This is consistent with experiments showing NR2B upregulation following dark rearing (Chen and Bear, 2007). Similarly, blocking NMDA receptor function by acute knockout prevents the increase in mEPSC levels seen in visual cortex L2/3 cells (Rodriguez et al., 2019). This type of L2/3 NMDA-dependent mechanism seems to be similar to the one we describe for layer 5 IB cells, since NMDA receptors induce plasticity via calcium and CaMKII-autophosphorylation (to produce LTP) and also promote new spine survival and enlargement of pre-existing spines (Giese et al., 1998; Hardingham et al., 2003; Seaton et al., 2020).

However, the same mechanism is not triggered in layer 5 RS cells during DE; they do not show potentiation and their homeostatic recovery is not CaMKII dependent. The same can be said for RS cells in barrel cortex (Greenhill et al., 2015). The situation appears to be more complicated for layer 2/3 cells, which are mostly RS (Mason and Larkman, 1990) and show both Hebbian and synaptic scaling properties in visual (Ranson et al., 2012) and somatosensory cortex (Glazewski et al., 2017).

Relationship between structural and functional changes

In this study, we discovered a number of previously unreported differences between IB and RS cells. While IB cells showed mature synaptic properties at the start of the critical period, layer 5 RS cells matured during the critical period. Furthermore, the two cell types showed different spine morphologies, with IB cells having a greater proportion of mushroom spines and RS cells having a higher proportion of long thin spines.

In most cases we did not find clear correlations between structural and functional changes; however, in the case of DE we found that the structural changes in IB cells preceded the functional changes; spines enlarged after just 12 h of DE, whereas the mEPSC amplitude was, on average, unchanged at this time point. The functional increase in mEPSC amplitude was observed at 3 days, by which time the spine size had fallen back from its peak (at 12 h). These observations are consistent with the findings of two separate studies, each of which reported one of the effects. First, Barnes et al. (2017) detected an increase in spine head size just 8 h after eye enucleation (Barnes et al., 2017). Second, in a separate study, recordings from layer 5 in animals receiving retinal lesions showed no increase in mEPSC amplitude at 6 and 18 h; instead, increases only became apparent after 24 h (Keck et al., 2013). Taken together with this study, it would appear that an increase in

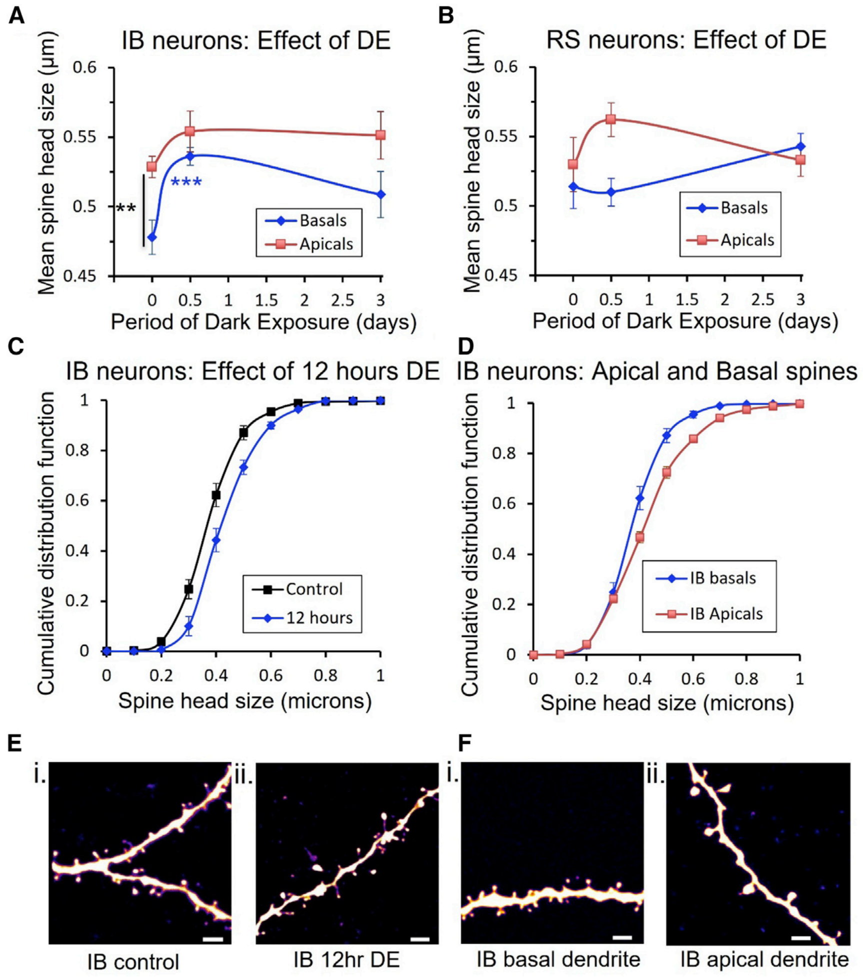

Figure 6. Changes in spine head diameter following dark exposure

(A) In IB cells, spine head size increases for spines on basal dendrites after 12 h of dark exposure (blue).

(B) Significant changes in spine head size could not be detected in RS cells following DE.

(C) CDFs for basal dendritic spines from IB cells show that the increase in IB spine head size after 12 h DE (blue) is uniform across the population of spines.

(D) Spine head sizes show a scaling difference between apical (red) and basal (blue) spines from IB cells.

(E) Example dendritic spines on basal dendrites of IB neurons from a control animal (Ei); basal dendrites of IB neurons from an animal after 12 h DE (Eii).

(F) Basal dendrites of an IB neuron from a control animal (Fi), (apical dendrite of an IB neuron from a control animal (Fii). Scale bar, 2 µm (in spines images). Data points represent means ± SEM. See also Figure S6.

spine head size precedes a functional increase in mEPSC amplitude.

Studies in hippocampal cultured cells have also demonstrated that the first change following NMDA receptor activation is an increase in spine size, which precedes an increase in AMPA receptor surface expression (Lin et al., 2004). Both changes in structure and function are controlled by CaMKII, as demonstrated by *in vitro* (Araki et al., 2015) and *in vivo* studies (Glazewski et al., 1996; Seaton et al., 2020). Crucially, we find that DE does not produce functional changes in mice lacking CaMKII-autophosphorylation, which indicates that the spine enlargement mechanisms described in these methodologically different studies (Araki et al., 2015; Keck et al., 2013; Lin et al., 2004) are indeed related to our observations in IB cells.

Limitations of the study

One limitation of this study is that it only comprises *in vitro* data. While previous studies have noted that changes in sensory responses due to sensory deprivation are strongly correlated with changes in mEPSC amplitude (Greenhill et al., 2015), it remains possible that cortical circuit changes, for example, in inhibitory cells, may also play a part in shaping sensory responses under deprivation conditions. *In vivo* recordings of IB and RS cell responses would resolve this question.

A second limitation arises from not having compared dendritic spine plasticity by tracking individual spines across time, but instead comparing spine properties in different cohorts of animals. This means that some structure-function relationships are likely hidden in the variability of spine population dynamics in our dataset. While the structure-function relationships we did find are likely to be extremely robust features of plasticity, those we did not find, for example, any spine changes related to depression, remain to be identified.

STAR★METHODS

Detailed methods are provided in the online version of this paper and include the following:

- KEY RESOURCES TABLE
- RESOURCE AVAILABILITY
  - Lead contact
  - Materials availability
  - Data and code availability
- EXPERIMENTAL MODEL AND SUBJECT DETAILS
  - Mouse
- METHOD DETAILS
  - Visual deprivation
  - Surgeries for RetroBeads injections
  - *In vitro* electrophysiology
  - Dendritic spine imaging

CellPress
OPEN ACCESS

- QUANTIFICATION AND STATISTICAL ANALYSIS
  - Morphological analysis
  - Measurements of spine morphology
  - Data analysis and statistics

SUPPLEMENTAL INFORMATION

Supplemental information can be found online at https://doi.org/10.1016/j.celrep.2022.110892.

ACKNOWLEDGMENTS

The authors thank the MRC for funding this research (MR/N003896/1), David Szymkowski and INmune Bio for donating the XPro1595, Frank Sengpiel for critical reading of the text, and Stephanie Bagstaff for technical assistance.

AUTHOR CONTRIBUTIONS

K.F. conceived the research project and obtained funding. A.P. and N.H. conducted the experiments. K.F., N.H., and A.P. designed the research, analyzed the data, and wrote the paper.

DECLARATION OF INTERESTS

The authors declare no competing interests.

Received: February 14, 2022
Revised: April 5, 2022
Accepted: May 9, 2022
Published: May 31, 2022

REFERENCES

Agmon, A., and Connors, B.W. (1992). Correlation between intrinsic firing patterns and thalamocortical synaptic responses of neurons in mouse barrel cortex. J. Neurosci. *12*, 319–329. https://doi.org/10.1523/jneurosci.12-01-00319.1992.

Araki, T., Cruz-Silva, R., Tejima, S., Takeuchi, K., Hayashi, T., Inukai, S., Noguchi, T., Tanioka, A., Kawaguchi, T., Terrones, M., and Endo, M. (2015). Molecular dynamics study of carbon nanotubes/polyamide reverse osmosis membranes: polymerization, structure, and hydration. ACS Appl. Mater. Inter. *7*, 24566–24575. https://doi.org/10.1021/acsami.5b06248.

Barnes, S.J., Franzoni, E., Jacobsen, R.I., Erdelyi, F., Szabo, G., Clopath, C., Keller, G.B., and Keck, T. (2017). Deprivation-induced homeostatic spine scaling in vivo is localized to dendritic branches that have undergone recent spine loss. Neuron *96*, 871–882.e5. https://doi.org/10.1016/j.neuron.2017.09.052.

Bridi, M.C.D., de Pasquale, R., Lantz, C.L., Gu, Y., Borrell, A., Choi, S.Y., He, K., Tran, T., Hong, S.Z., Dykman, A., et al. (2018). Two distinct mechanisms for experience-dependent homeostasis. Nat. Neurosci. *21*, 843–850. https://doi.org/10.1038/s41593-018-0150-0.

Chang, J.Y., Parra-Bueno, P., Laviv, T., Szatmari, E.M., Lee, S.J.R., and Yasuda, R. (2017). CaMKII autophosphorylation is necessary for optimal integration of Ca(2+) signals during LTP induction, but not maintenance. Neuron *94*, 800–808.e4. https://doi.org/10.1016/j.neuron.2017.04.041.

Chen, W.S., and Bear, M.F. (2007). Activity-dependent regulation of NR2B translation contributes to metaplasticity in mouse visual cortex. Neuropharmacology *52*, 200–214. https://doi.org/10.1016/j.neuropharm.2006.07.003.

Cooke, S.F., and Bear, M.F. (2010). Visual experience induces long-term potentiation in the primary visual cortex. J. Neurosci. *30*, 16304–16313. https://doi.org/10.1523/JNEUROSCI.4333-10.2010.

Daw, N.W., Gordon, B., Fox, K.D., Flavin, H.J., Kirsch, J.D., Beaver, C.J., Ji, Q.H., Reid, S.N., and Czepita, D. (1999). Injection of MK-801 affects ocular dominance shifts more than visual activity. J. Neurophysiol. *81*, 204–215. https://doi.org/10.1152/jn.1999.81.1.204.

Gao, W.J., and Zheng, Z.H. (2004). Target-specific differences in somatodendritic morphology of layer V pyramidal neurons in rat motor cortex. J. Comp. Neurol. *476*, 174–185. https://doi.org/10.1002/cne.20224.

Giese, K.P., Fedorov, N.B., Filipkowski, R.K., and Silva, A.J. (1998). Autophosphorylation at Thr286 of the alpha calcium-calmodulin kinase II in LTP and learning. Science *279*, 870–873. https://doi.org/10.1126/science.279.5352.870.

Glazewski, S., Chen, C.M., Silva, A., and Fox, K. (1996). Requirement for alpha-CaMKII in experience-dependent plasticity of the barrel cortex. Science *272*, 421–423. https://doi.org/10.1126/science.272.5260.421.

Glazewski, S., Greenhill, S., and Fox, K. (2017). Time-course and mechanisms of homeostatic plasticity in layers 2/3 and 5 of the barrel cortex. Philos. Trans. R. Soc. Lond. B Biol. Sci. *372*, 20160150. https://doi.org/10.1098/rstb.2016.0150.

Gordon, J.A., and Stryker, M.P. (1996). Experience-dependent plasticity of binocular responses in the primary visual cortex of the mouse. J. Neurosci. *16*, 3274–3286. https://doi.org/10.1523/jneurosci.16-10-03274.1996.

Greenhill, S.D., Ranson, A., and Fox, K. (2015). Hebbian and homeostatic plasticity mechanisms in regular spiking and intrinsic bursting cells of cortical layer 5. Neuron *88*, 539–552. https://doi.org/10.1016/j.neuron.2015.09.025.

Gu, Q.A., Bear, M.F., and Singer, W. (1989). Blockade of NMDA-receptors prevents ocularity changes in kitten visual cortex after reversed monocular deprivation. Brain Res. Dev. Brain Res. *47*, 281–288. https://doi.org/10.1016/0165-3806(89)90183-1.

Hardingham, N., Glazewski, S., Pakhotin, P., Mizuno, K., Chapman, P.F., Giese, K.P., and Fox, K. (2003). Neocortical long-term potentiation and experience-dependent synaptic plasticity require alpha-calcium/calmodulin-dependent protein kinase II autophosphorylation. J. Neurosci. *23*, 4428–4436. https://doi.org/10.1523/jneurosci.23-11-04428.2003.

Hattox, A.M., and Nelson, S.B. (2007). Layer V neurons in mouse cortex projecting to different targets have distinct physiological properties. J. Neurophysiol. *98*, 3330–3340. https://doi.org/10.1152/jn.00397.2007.

Heynen, A.J., Yoon, B.J., Liu, C.H., Chung, H.J., Huganir, R.L., and Bear, M.F. (2003). Molecular mechanism for loss of visual cortical responsiveness following brief monocular deprivation. Nat. Neurosci. *6*, 854–862. https://doi.org/10.1038/nn1100.

Hooks, B.M., and Chen, C. (2020). Circuitry underlying experience-dependent plasticity in the mouse visual system. Neuron *107*, 986–987. https://doi.org/10.1016/j.neuron.2020.08.004.

Jacob, V., Mitani, A., Toyoizumi, T., and Fox, K. (2017). Whisker row deprivation affects the flow of sensory information through rat barrel cortex. J. Neurophysiol. *117*, 4–17. https://doi.org/10.1152/jn.00289.2016.

Kaneko, M., Stellwagen, D., Malenka, R.C., and Stryker, M.P. (2008). Tumor necrosis factor-alpha mediates one component of competitive, experience-dependent plasticity in developing visual cortex. Neuron *58*, 673–680. https://doi.org/10.1016/j.neuron.2008.04.023.

Kasper, E.M., Larkman, A.U., Lubke, J., and Blakemore, C. (1994). Pyramidal neurons in layer 5 of the rat visual cortex. I. Correlation among cell morphology, intrinsic electrophysiological properties, and axon targets. J. Comp. Neurol. *339*, 459–474. https://doi.org/10.1002/cne.903390402.

Keck, T., Keller, G.B., Jacobsen, R.I., Eysel, U.T., Bonhoeffer, T., and Hubener, M. (2013). Synaptic scaling and homeostatic plasticity in the mouse visual cortex in vivo. Neuron *80*, 327–334. https://doi.org/10.1016/j.neuron.2013.08.018.

Kim, E.J., Juavinett, A.L., Kyubwa, E.M., Jacobs, M.W., and Callaway, E.M. (2015). Three types of cortical layer 5 neurons that differ in brain-wide connectivity and function. Neuron *88*, 1253–1267. https://doi.org/10.1016/j.neuron.2015.11.002.

Kirkwood, A., Silva, A., and Bear, M.F. (1997). Age-dependent decrease of synaptic plasticity in the neocortex of αCaMKII mutant mice. Proc. Natl. Acad. Sci. U S A *94*, 3380–3383. https://doi.org/10.1073/pnas.94.7.3380.

Lake, B.B., Ai, R., Kaeser, G.E., Salathia, N.S., Yung, Y.C., Liu, R., Wildberg, A., Gao, D., Fung, H.L., Chen, S., et al. (2016). Neuronal subtypes and diversity

revealed by single-nucleus RNA sequencing of the human brain. Science *352*, 1586–1590. https://doi.org/10.1126/science.aaf1204.

Lin, H., Huganir, R., and Liao, D. (2004). Temporal dynamics of NMDA receptor-induced changes in spine morphology and AMPA receptor recruitment to spines. Biochem. Biophys. Res. Commun. *316*, 501–511. https://doi.org/10.1016/j.bbrc.2004.02.086.

Lodato, S., and Arlotta, P. (2015). Generating neuronal diversity in the mammalian cerebral cortex. Annu. Rev. Cel. Dev. Biol. *31*, 699–720. https://doi.org/10.1146/annurev-cellbio-100814-125353.

Loewenstein, Y., Kuras, A., and Rumpel, S. (2011). Multiplicative dynamics underlie the emergence of the log-normal distribution of spine sizes in the neocortex in vivo. J. Neurosci. *31*, 9481–9488. https://doi.org/10.1523/jneurosci.6130-10.2011.

Magee, J.C., and Cook, E.P. (2000). Somatic EPSP amplitude is independent of synapse location in hippocampal pyramidal neurons. Nat. Neurosci. *3*, 895–903. https://doi.org/10.1038/78800.

Mason, A., and Larkman, A. (1990). Correlations between morphology and electrophysiology of pyramidal neurons in slices of rat visual cortex. II. Electrophysiology. J. Neurosci. *10*, 1415–1428. https://doi.org/10.1523/jneurosci.10-05-01415.1990.

Medini, P. (2011a). Cell-type-specific sub- and suprathreshold receptive fields of layer 4 and layer 2/3 pyramids in rat primary visual cortex. Neuroscience *190*, 112–126. https://doi.org/10.1016/j.neuroscience.2011.05.026.

Medini, P. (2011b). Layer- and cell-type-specific subthreshold and suprathreshold effects of long-term monocular deprivation in rat visual cortex. J. Neurosci. *31*, 17134–17148. https://doi.org/10.1523/JNEUROSCI.2951-11.2011.

Pandey, A., and Sikdar, S.K. (2014). Depression biased non-Hebbian spike-timing-dependent synaptic plasticity in the rat subiculum. J. Physiol. *592*, 3537–3557. https://doi.org/10.1113/jphysiol.2014.273367.

Paxinos, G., and Franklin, K.B.J. (2008). The Mouse Brain in Stereotaxic Coordinates, 3rd Edition (Academic Press).

Ranson, A., Cheetham, C.E.J., Fox, K., and Sengpiel, F. (2012). Homeostatic plasticity mechanisms are required for juvenile, but not adult, ocular dominance plasticity. Proc. Natl. Acad. Sci. U S A *109*, 1311–1316. https://doi.org/10.1073/pnas.1112204109.

Rodriguez, G., Mesik, L., Gao, M., Parkins, S., Saha, R., and Lee, H.K. (2019). Disruption of NMDAR function prevents normal experience-dependent homeostatic synaptic plasticity in mouse primary visual cortex. J. Neurosci. *39*, 7664–7673. https://doi.org/10.1523/JNEUROSCI.2117-18.2019.

Schubert, D., Kotter, R., and Staiger, J.F. (2007). Mapping functional connectivity in barrel-related columns reveals layer- and cell type-specific microcircuits. Brain Struct. Funct. *212*, 107–119. https://doi.org/10.1007/s00429-007-0147-z.

Schubert, D., Kotter, R., Luhmann, H.J., and Staiger, J.F. (2006). Morphology, electrophysiology and functional input connectivity of pyramidal neurons characterizes a genuine layer va in the primary somatosensory cortex. Cereb. Cortex *16*, 223–236. https://doi.org/10.1093/cercor/bhi100.

Seaton, G., Hodges, G., de Haan, A., Grewal, A., Pandey, A., Kasai, H., and Fox, K. (2020). Dual-Component structural plasticity mediated by αCaMKII autophosphorylation on basal dendrites of cortical layer 2/3 neurones. J. Neurosci. *40*, 2228–2245. https://doi.org/10.1523/jneurosci.2297-19.2020.

Steed, P.M., Tansey, M.G., Zalevsky, J., Zhukovsky, E.A., Desjarlais, J.R., Szymkowski, D.E., Abbott, C., Carmichael, D., Chan, C., Cherry, L., et al. (2003). Inactivation of TNF signaling by rationally designed dominant-negative TNF variants. Science *301*, 1895–1898. https://doi.org/10.1126/science.1081297.

Stellwagen, D., and Malenka, R.C. (2006). Synaptic scaling mediated by glial TNF-α. Nature *440*, 1054–1059. https://doi.org/10.1038/nature04671.

Toyoizumi, T., Kaneko, M., Stryker, M.P., and Miller, K.D. (2014). Modeling the dynamic interaction of Hebbian and homeostatic plasticity. Neuron *84*, 497–510. https://doi.org/10.1016/j.neuron.2014.09.036.

Turrigiano, G.G., Leslie, K.R., Desai, N.S., Rutherford, L.C., and Nelson, S.B. (1998). Activity-dependent scaling of quantal amplitude in neocortical neurons. Nature *391*, 892–896. https://doi.org/10.1038/36103.

Wiesel, T.N., and Hubel, D.H. (1963). Single-cell responses in striate cortex of kittens deprived of vision in one eye. J. Neurophysiol. *26*, 1003–1017. https://doi.org/10.1152/jn.1963.26.6.1003.

CellPress
OPEN ACCESS

STAR★METHODS

KEY RESOURCES TABLE

REAGENT or RESOURCE	SOURCE	IDENTIFIER
**Experimental models: Organisms/strains**		
C57Bl/6J wild type mice	Charles River, UK	RRID: IMSR_JAX:000664
transgenic homozygous αCaMKII^{T286A} mutant mice	A gift from Silva lab at UCLA http://www.silvalab.org/index.html	αCaMKII^{T286A}
**Chemicals, peptides, and recombinant proteins**		
XPro1595	INmune Bio Inc, USA	XPro1595
6/0 non-absorbable sutures	Mersilk-Ethicon, J&J	W529H
1.0% Chloramphenicol 1.0% w/w Eye ointment	Martindale Pharma	1.0% Chloramphenicol 1.0% w/w Eye ointment
Red RetroBeads	Lumafluor inc., NC, USA	Red Retrobeads™
Meloxicam	Boehringer Ingelheim	Metacam®
Dexamethasone	Northbrook Laboratories, UK	Colvasone 0.2% w/v
Lidocaine Hydrochloride injection 1%	Hamlen, UK	Lidocaine Hydrochloride
Sodium Chloride 0.9% w/v and Glucose 5% w/v Solution	Baxter Healthcare Ltd, UK	Sodium Chloride 0.9% w/v and Glucose 5% w/v Solution
Clark Borosilicate glass capillaries	Warner instruments, USA	GC100F-10
UMP3 UltraMicro Pump for cortical injections	World precision instruments	UMP3-4
CED Micro 1401 AD-converter	Cambridge Electronic Design Limited, UK	Micro1401
Multiclamp 700B amplifier	Molecular devices	Multiclamp 700B
Biocytin	Merck	576-19-2
Sodium chloride	Merck	S7653
Potassium chloride	Fisher scientific	P/4280/53
Calcium chloride	Merck	21115
Magnesium sulfate	Merck	434183
Sodium Bicarbonate	Fisher scientific	BP328-500
Sodium phosphate monobasic	Merck	S0751
Potassium D-gluconate	Merck	P4500
HEPES	Merck	H3375
Adenosine 5′-triphosphate magnesium salt (ATP Magnesium salt)	Merck	A9187
Guanosine 5′-triphosphate sodium salt hydrate (GTP Sodium salt)	Merck	G8877
D(+)-Glucose, ACS reagent, anhydrous	Thermo Fisher scientific	410950010
Choline chloride	Merck	C7527
Tetrodotoxin citrate	Hello Bio, UK	HB1035
DL-AP5	Hello Bio, UK	HB0251
Picrotoxin	Hello Bio, UK	HB0506
Paraformaldehyde	Merck	8.18715
Alexa Fluor 488 Streptavidin	Invitrogen	S11223
**Software and algorithms**		
Signal	Cambridge Electronic Design Limited, UK	Signal version 7
Spike2	Cambridge Electronic Design Limited, UK	Spike2 version 4
AxoGraph	Axograph.com	AxoGraph 1.7.6
Matlab	Mathworks	Matlab R2021b
Matlab graphing tools for plotting CDF from mEPSC amplitude and inter-event interval files	Github	https://doi.org/10.5281/zenodo.6482880
ImageJ	NIH, USA	ImageJ
JMP	SAS software, USA	JMP

RESOURCE AVAILABILITY

Lead contact

Further information and reasonable requests for data should be directed to and will be fulfilled by the lead contact, Kevin Fox (FoxKD@cardiff.ac.uk).

Materials availability

This study did not generate new unique reagents. All resources are commercially available except the CaMKII-t286a mice, which are currently being deposited at Jackson Labs.

Data and code availability

Data

All data reported in this paper will be shared by the lead contact Kevin Fox (FoxKD@cardiff.ac.uk) upon reasonable request.

Code

All original code has been deposited at Github and is publicly available as of the date of publication. DOIs are listed in the key resources table. The code comprises Matlab scripts for use with tabular mEPSC amplitude and interval data that will calculate optimal bin intervals, create cumulative frequency distributions, and plot them in graphical form. Example data in the format expected by the scripts are also deposited.

Any additional information required to reanalyze the data reported in this paper is available from the lead contact upon request.

EXPERIMENTAL MODEL AND SUBJECT DETAILS

Mouse

All the procedures were approved under the Animal (Scientific Procedures) Act 1986. A total of 268 mice were used in the study across different age groups, genotypes, deprivation time points, cell types, visual deprivation group (monocular deprivation or dark exposure) and treatment groups (XPro 1595 or saline) (see Table S2). All the mice used were either WT C57Bl/6J (RRID:IMSR_JAX:000664, acquired from Charles River, UK) or transgenic homozygous αCaMKII^{T286A} mutants on the C57Bl/6J background. The genotypes of the transgenic animals were determined by PCR analysis with DNA obtained from ear biopsies on postnatal day 21. Animals from both male and female sexes were used. The transgenic homozygous αCaMKII^{T286A} mutant mice have a point mutation at location 286 in αCamKII where Thr(T) is replaced with Alanine(A). This results in CamKII being unable to switch to its CaM-independent state. These mice have been shown to lack NMDAR dependent LTP and learning (Giese et al., 1998). The control mice were housed in standard housing conditions of 12 h light/dark cycle. All the animals used were between 26 and 38 days old. The age group of the population of animals used in each experiment is given in Table S2. Homozygous αCaMKII^{T286A} mutant mice were generated by breeding heterozygous αCaMKII^{T286A} mutant male and female mice. The data for DE of αCaMKII^{T286A} mutant mice were compared with data obtained from αCaMKII^{T286A} mutant mice of a similar age group but housed under standard housing conditions. An on-receptor binding variant of TNFα, XPro1595 (INmune Bio inc, USA) that forms heterotrimers with native soluble TNFα and prevents its interaction with the type 1 TNFα receptor (Steed et al., 2003) is used to neutralise TNFα. A subset of WT C57Bl/6J animals were treated with intraperitoneal injection of XPro-1595 at 1 mg per Kg of animal's body weight. The drug was diluted in saline so that the total liquid injection was not more than 500 microliters per animal. XPro-1595 or saline was injected at least 12hrs before the electrophysiological recordings or the dark exposure starting point. In the case of 5d DE, XPro-1595 was injected twice - 12hr before the start of DE and on third day of DE. Control animals corresponding to XPro-1595 injected DE animals were injected with a similar volume of saline following a similar injection schedule.

METHOD DETAILS

Visual deprivation

Littermates were randomly allocated to the control or experimental groups, irrespective of sex. All the animals were housed under standard conditions up to the start of dark exposure or monocular deprivation. Visual deprivation of the mice was started at the age of 26–31 days. Animals deprived for different periods of time were therefore different ages when they were recorded. For example, the animals with 10 days of MD were older than animals with 12 h or 3days of MD, as the deprivation starting point was similar. We therefore compared animals undergoing deprivation with controls matched to their age on the day of recording. For dark exposure experiments, two mice were housed together in the dark room for a given duration. For monocular deprivation experiments, the eyelid of one eye was sutured under brief isoflurane anesthesia (6/0 non-absorbable sutures, Mersilk-Ethicon, J&J) and topical antibiotics applied (1.0% Chloramphenicol 1.0% w/w Eye ointment, Martindale Pharma). The sutures were checked every day to monitor constant closure and the experiment was terminated if gaps developed between eyelids. After eyelid suture the animals were housed with a littermate.

Surgeries for RetroBeads injections

Both male and female WT C57Bl/6J animals (5-week-old) were used for tracing experiments. These animals were housed under standard housing conditions without any visual deprivation. For labeling neurons projecting to various targets, red retrograde latex beads (RetroBeads, Lumafluor inc., NC, USA, excitation max = 530 nm, emission max = 590 nm) were injected in the putative target sites. The beads were diluted 4X from the 10% w/v stock solution provided by the manufacturer. For labeling contralateral visual cortex projecting neurons, retrobeads (100 nL at the flow rate of 20 nL per minute) were injected using glass needles with a tip diameter of 20-50 μm using an ultra-micropump (WPI). The injections were performed at two locations in visual cortex layer 5 (−3.8 mm AP from Bregma, 2.5 mm ML from midline, 0.7 mm DV from Pia and −3.5 mm AP from Bregma, 2.3 mm ML from midline, 0.7 mm DV from Pia). To label neurons projecting to the Superior colliculus, retrobeads were injected at (−3.9 mm AP from Bregma, 0.1 mm ML from midline, 2.2 mm DV from Pia) with the injection needle tilted at 25^0 from the midline. The injection needle was held at the injection location for 10 min after injection to make sure there was no backflow of the beads into superficial layers.

In vitro electrophysiology

Mice were killed by decapitation. The brain was quickly removed and immediately placed in ice cold slicing solution (in mM: 108 Choline-Cl, 3 KCl, 26 $NaHCO_3$, 1.25 NaH_2PO_4, 25 D-glucose, 3 NaPyruvate, 1 $CaCl_2$, 6 $MgSO_4$, 285 mOsm, bubbled with 95% O_2 5% CO_2). 350 μm thick coronal slices were cut in ice cold slicing solution using a vibrating microtome (Microm HM650V, Thermofisher). Slices were then transferred to a holding chamber containing normal ACSF (in mM: 119 NaCl, 3.5 KCl, 26 $NaHCO_3$, 1 NaH_2PO_4, 2 $CaCl_2$, 1 $MgSO_4$, 10 D-glucose, 300 mOsm bubbled with 95% O_2 5% CO_2). Slices were incubated at 37°C for 30 min and then returned to room temperature for 30 min before recordings, which were performed at room temperature. The area of binocular visual cortex was identified using mouse brain atlas by Paxinos (Paxinos and Franklin, 2008). In case of monocular deprivation, we recorded mEPSCs from the hemisphere contralateral to the deprived eye within the binocular zone. Layers 5A and 5B were identified under bright field illumination using differential interference contrast on an Olympus BX50WI microscope, guided by the neuronal density and morphology of the cells. Whole cell voltage/current clamp recordings were performed using borosilicate glass electrodes (4-7 MΩ) filled with a potassium-gluconate based solution (in mM: 110 K-gluconate, 10 KCl, 2 $MgCl_2$, 2 Na_2ATP, 0.03 Na_2GTP, 10 HEPES, pH 7.3, 270 mOsm). In a subset of experiments, biocytin (Sigma, UK) was added to the electrode filling solution at a concentration of 5 mg/mL to enable cells to be morphologically characterised.

Pyramidal neurons were classified into IB and RS neurons based on their spiking properties at threshold. Briefly, the neurons were classified as IB neurons if the first action potentials fired by the neuron at threshold were in the form of one or multiple bursts of action potentials, otherwise the neurons were classified as RS neurons if only single action potentials were observed. A burst of action potentials was defined as at least 2 action potentials firing at a frequency higher than 100 Hz as described earlier (Pandey and Sikdar, 2014). The experimenter was blind to the type of neurons to be sampled from a given mouse, as neurone types cannot be identified before electrophysiological recordings are made under DIC microscope without detailed morphological information. Once a successful recording was established that satisfied the essential criteria of a stable recording, mEPSCs from the neurone were recorded and the data were included in the final analysis, irrespective of the neuronal subtype. The recordings were not made if a cell was electrophysiologically identified as an interneurone, as they are not part of the present study. The essential criteria for a recording to be included in the final analysis were a resting membrane potential more hyperpolarized than −60 mV, a series resistance of less than 20MΩ and not changing by more than 20% over the recording and a stable resting membrane potential. After the characterisation of active properties of the neurons, slices were perfused with ACSF containing a cocktail of drugs consisting of 50 μM DL-AP5, 10μM picrotoxin and 1μM TTX. The mEPSCs were recorded in the voltage clamp configuration. Recordings were made using an Axon Multiclamp 700B, digitized with a CED Micro 1401 controlled with CED Signal software. Miniature EPSCs were analyzed with Axograph software using a template-matching method (see data analysis in the quantification and statistical analysis section).

Dendritic spine imaging

After electrophysiological recordings in a subset of animals, slices containing biocytin filled neurons were fixed overnight at 4°C in 0.1M phosphate buffered saline (PBS, pH 7.3) containing 4% paraformaldehyde. Thereafter, slices were washed with 0.1M PBS and incubated in 0.1M PBS supplemented with 1% Triton X-100 and 0.2% streptavidin Alexa Fluor 488 conjugate (Invitrogen) at 4°C for 18hrs. After further washing with 0.1M PBS, cells were imaged under a 2-photon microscope (Prarie Systems, USA) using a mode-locked Ti:sapphire laser (Chameleon Vision S, Coherent) to generate two-photon excitation (900 nm), and emission wavelengths were band-passed between 525 and 570 nm (including an IR filter in the light path). A 10x objective lens was used to image gross cell morphology. For detailed dendritic and spine morphology a 60X objective lens was used and images were digitally magnified by 4.02 times. The complete dendritic morphology was measured for each filled neurone, while spine morphology was recorded for a subset of the dendrites, which fully described the neurone's dendritic arborisations (basal dendrites, apical obliques and apical tuft). The experimenter acquiring the images was not aware of the sensory manipulations that the animals had gone through. All the imaged spines were located on secondary or tertiary dendritic branches. Image stacks were collapsed into z-stacks (Image J) which in turn were stitched together (Photoshop, Adobe) to produce a detailed 2D dendritic profile of the neurone (Figure 1A). The distance

of the soma from the pia, length of apical dendrites, sites of bifurcation and the horizontal spread of the dendrites were all measured using Image J (NIH, USA).

QUANTIFICATION AND STATISTICAL ANALYSIS

Morphological analysis

The neurones' dendritic fields were analyzed using Sholl analysis. We counted the number of occasions that the dendrites crossed the Sholl shells (radial interval = 30 μm) at increasing distance from the soma. The Sholl shells were centered at the soma for basal and apical oblique dendrites, while for the apical tuft they were centered at the main bifurcation of the apical dendrite. We also measured the distance of the cell bodies from the pial surface, the length of the apical dendrites and the distance of apical bifurcation from the soma.

Measurements of spine morphology

Dendritic spines were analyzed as described earlier (Seaton et al., 2020). Image stacks were first deconvolved using Fiji Deconvolution Lab plugin and the spines were measured using ImageJ (NIH, USA). Only structures that were protruding at least 0.4 μm from the dendritic branches were counted as spines. Spines were classified based on head size, neck width, and neck length measurements. Briefly, spines with head size: neck width ratio >1.15 and a neck length <0.9 μm were classified as mushroom spines, while thin spines had head size: neck width ratio >1.15 and a neck length >0.9 μm. Stubby spines had a neck length <0.9 μm and a head size: neck width ratio <1.15. We also saw a smaller number of filopodia, which have neck length >0.9 μm while head size: neck width ratio <1.15. Filopodia were not included in the spine analysis unless stated, as in the spine classification sections. Spines were analyzed from the most planar sections of the branches. At least 60 spines were measured from each type of dendrite (apical, oblique, or basal) for each neurone. For the spine density analyses the dendritic length was measured by following the curves of the branches using the segmented line tool of imageJ. Since the percentage of filopodia in the total spine population was very low, filopodia were excluded from the final analysis except for the quantification of spine types (as detailed in the results section). The analyser was blind to the animals' treatments that the spines referred to.

Data analysis and statistics

The mEPSCs were analyzed using Axograph X (Axograph.com), using the template matching procedure and then visually corrected for errors. Data were sampled for at least 5 min. Recording periods of 2–5 min were analyzed to extract at least 100 events from each neurone. If more than 100 events were extracted from a neurone, only the first 100 events were used for analysis. The number of events used across the neurons were kept constant to balance the weight of each neurone in the population data. Data were analyzed by comparing average mEPSC amplitudes across cell types, visual deprivation groups, genotype, treatment group (XPro1595) and deprivation timepoints using ANOVA followed by post-hoc *t*-tests where effects were detected (JMP, SAS software). Linear regression was used to test the strength and statistical significance of correlations. If the data was not normally distributed non-parametric statistics were used. Spine head size data was found to be log-normally distributed, and log transformed before applying parametric statistical methods. Chi-squared tests were used to test the differences in spine-types between cell-types and dendritic branch locations. In those cases where a cumulative frequency distribution was plotted, the bin width was determined using the Freedman-Diaconis rule. Statistical significance was indicated by $p < 0.05$ and lack of significance for $p \geq 0.05$ (actual p values are indicated at the relevant locations in the text, e.g. $p > 0.05$, $p < 0.05$, $p < 0.03$, $p < 0.02$, $p < 0.01$, $p < 0.005$, $p < 0.003$, $p < 0.002$, $p < 0.001$, $p < 0.0002$, $p < 0.0001$). Matlab, R and Sigma Plot software were used for data analysis and plotting graphs.

Cell Reports, Volume *39*

Supplemental information

Differentiation of Hebbian
and homeostatic plasticity mechanisms
within layer 5 visual cortex neurons

Anurag Pandey, Neil Hardingham, and Kevin Fox

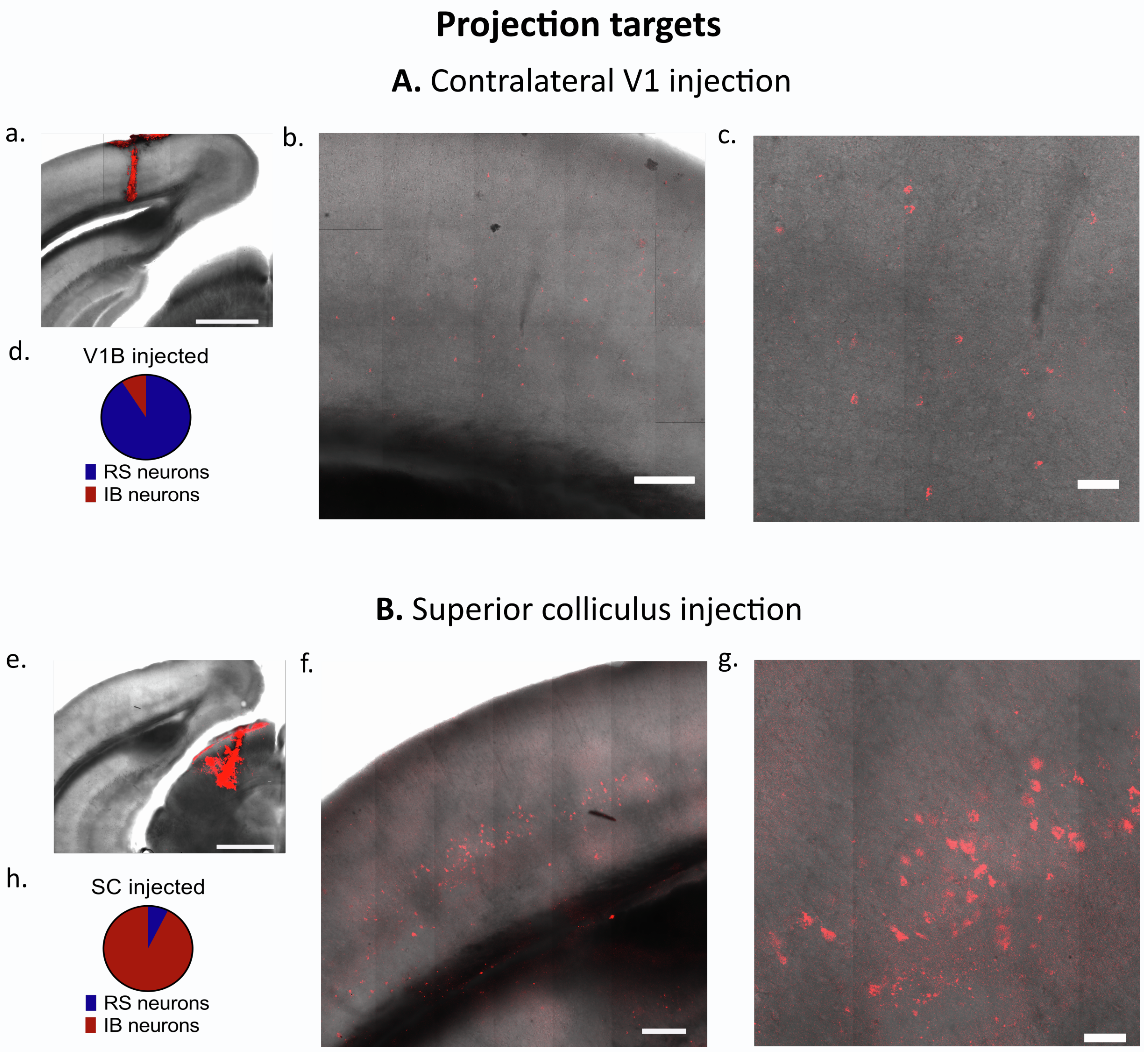

Figure S1. Coronal sections showing different projection targets of RS and IB neurons (related to Figure 1).

A. RS neurons project to contralateral visual cortex, while **B.** IB neurons project to superior colliculus (SC). **a.** Injection of retrobeads in V1b. Scale bar 1.00 mm **b.** Labelled neurons are found in all layers except layer I of visual cortex in the contralateral hemisphere. Scale bar 200µm. **c.** Magnified portion of a small area from panel b. Scale bar 50µm **d.** Pie chart representing proportion of RS and IB neurons among neurons projecting to contralateral visual cortex. **e.** Injection of retrobeads in superior colliculus. Scale bar 1.00 mm. **f.** Labelled neurons are mostly present in layer 5 of ipsilateral visual cortex. Scale bar 200µm. **g.** Magnified portion of a small area from panel f. Scale bar 50µm. **h.** Pie chart representing proportion of RS and IB neurons among neurons projecting to superior colliculus.

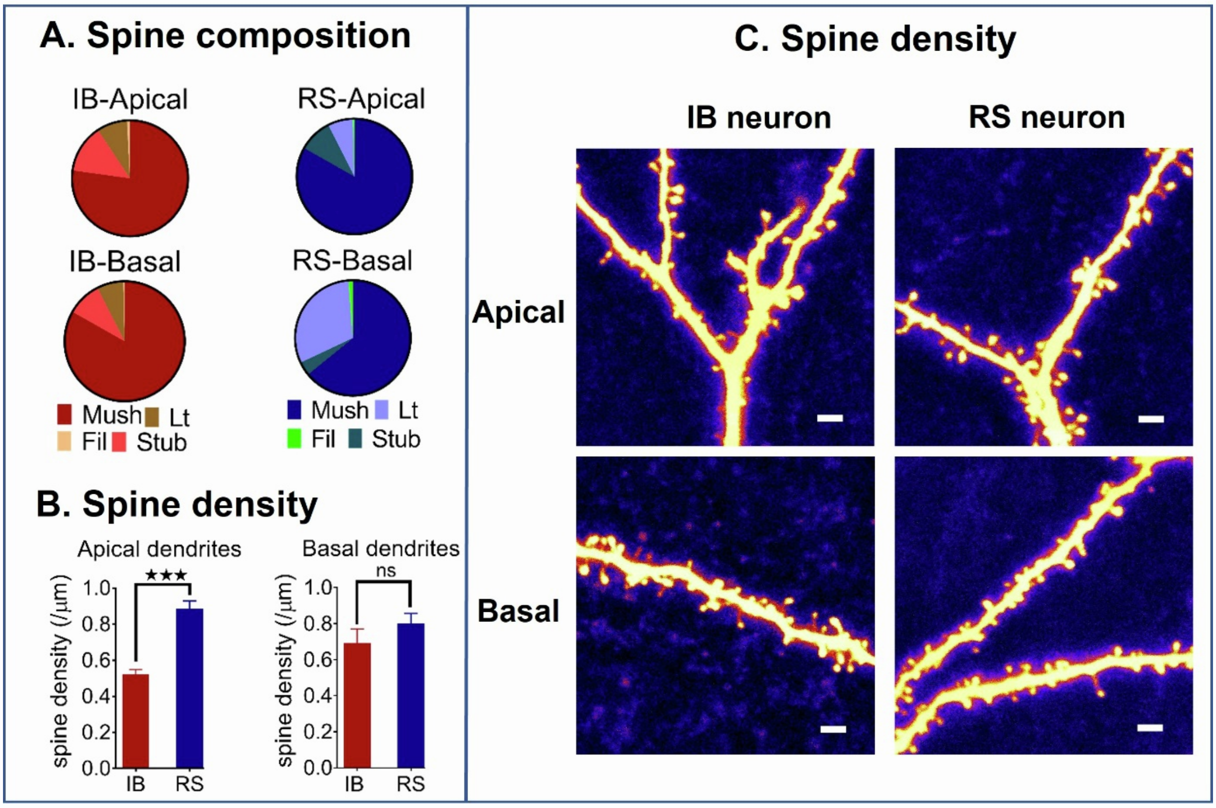

Figure S2. RS and IB neurons have different spine composition and density (related to Figure 1).

A. Pie charts showing basal dendrites of RS neurons have more long thin spines than the basal dendrites of IB neurons, while basal dendrites of IB neurons have more mushroom spines than basal dendrites of RS neurons (Mush – Mushroom spines, Lt- Long thin spines, Fil- Filopodia, Stub- Stubby spines). **B.** Apical dendrites of RS neurons have higher spine density than apical dendrites of IB neurons ($t_{(19)}$=7.26, p<0.001) while there is no difference in the spine density of basal dendrites of RS and IB cells. **C.** Representative images showing the above observations, scale bars – 2 µm.

*** p<0.001, ns not significant α=0.05. Histograms show means + SEM.

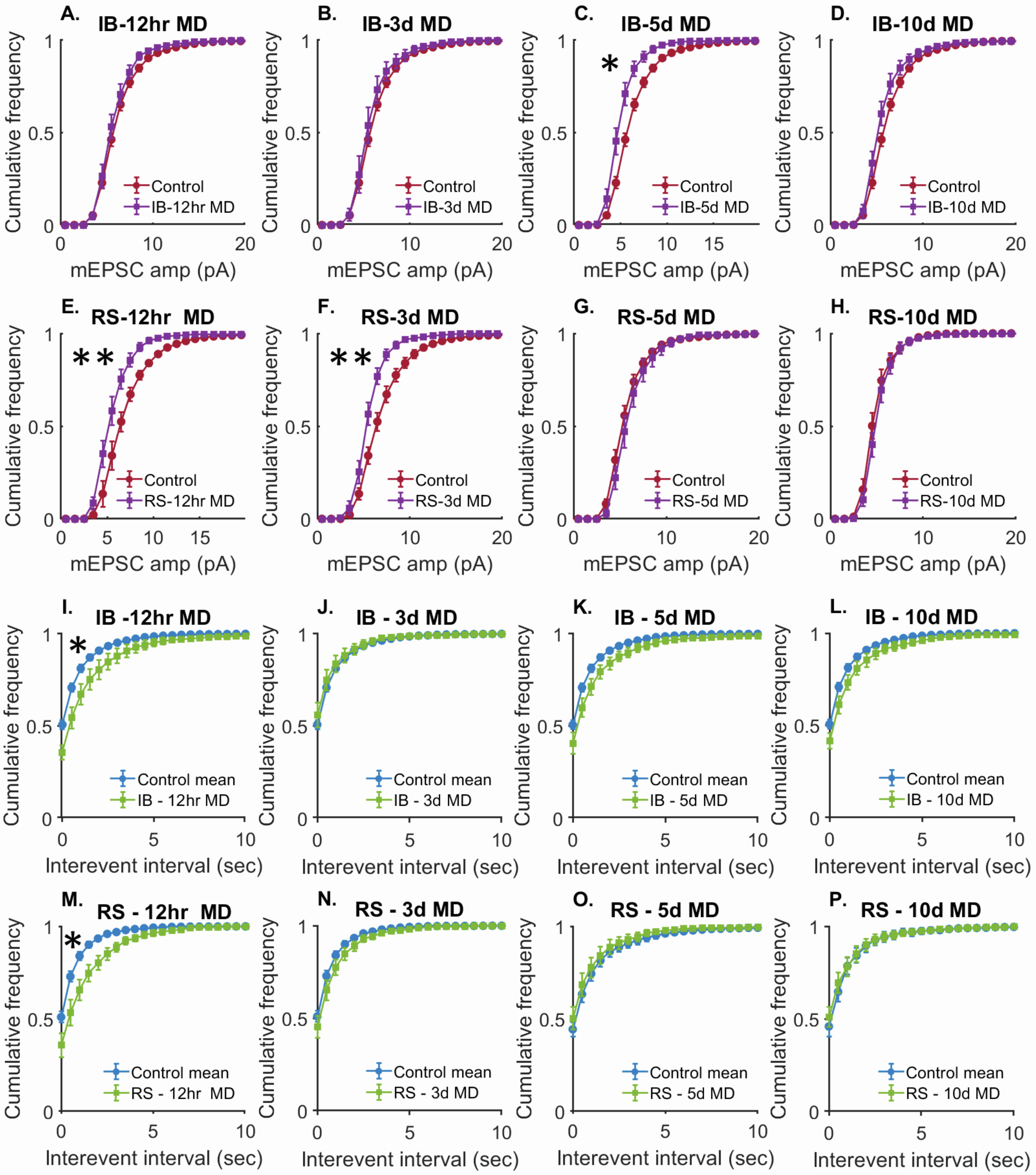

Figure S3. Effect of monocular deprivation on mEPSC amplitudes and inter-event intervals (related to Figure 3).

A-H: mEPSC amplitudes plotted in cumulative distribution functions (cdf). Control purple and MD maroon for IB (A-D) and RS cells (E-H). **A-D:** mEPSC amplitudes of IB neurons show reduction in mEPSC amplitudes after 5d MD, recovering to baseline after 10d of MD. **E-H:** mEPSC amplitudes of RS neurons show reduction after 12hr and 3d MD recovering to control on 5d MD. Bin interval-1pA.

I-L: mEPSC inter-event intervals plotted in cumulative distribution functions (cdf). Control blue and MD green for IB (I-L) and RS cells (M-P). **I-L**: Inter-event intervals increase for IB cells after 12 hours and recover after 3 days. **M-P:** RS neurons also show increase in mEPSC inter-event interval after 12 hours, followed by recovery at 3 days. Bin interval-0.5sec. *p<0.05, ** p<0.01. Plotted points are means ± SEM.

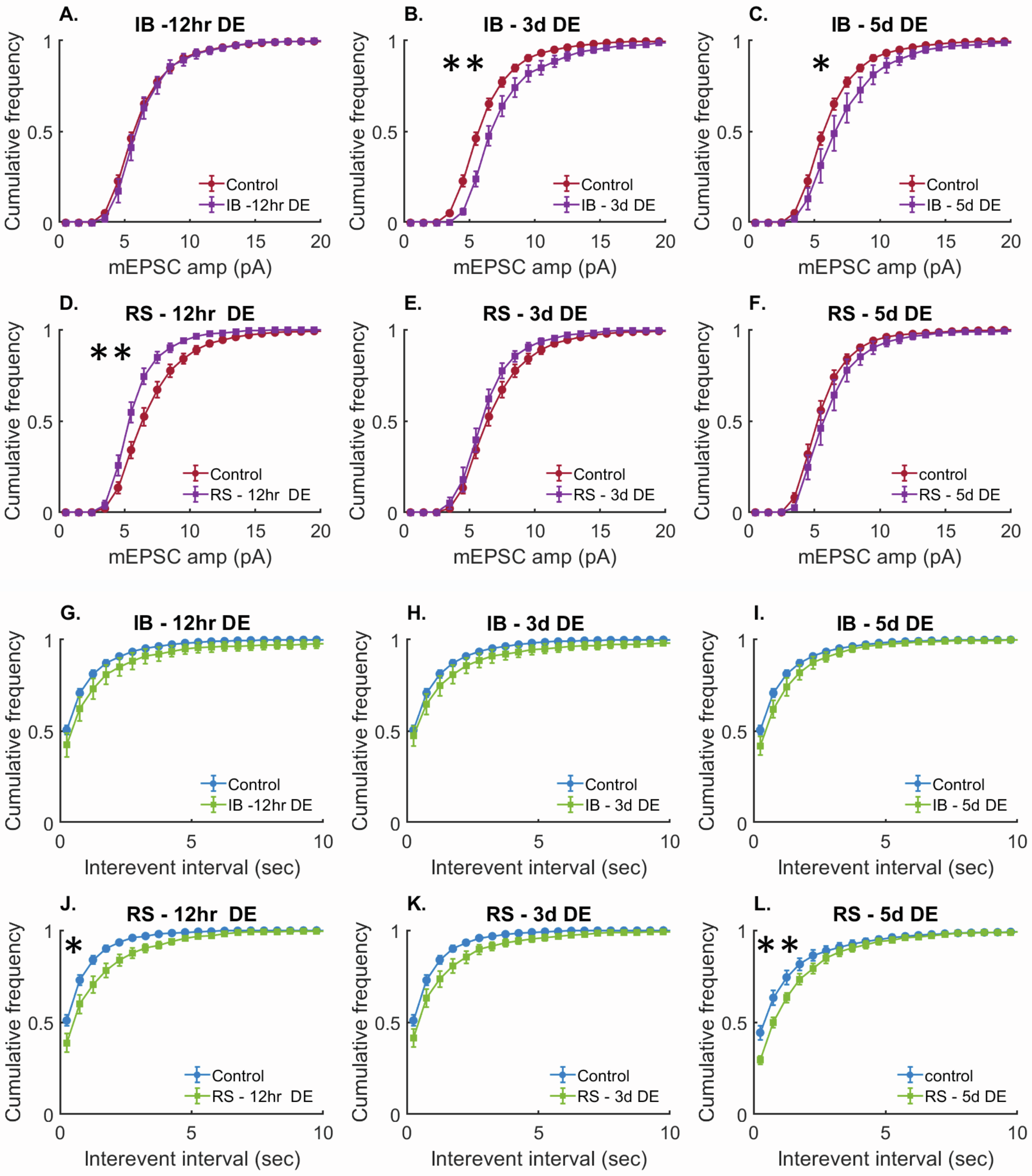

Figure S4. Effect of dark-exposure on mEPSC amplitudes and inter-event intervals (related to Figure 4).

A-F: mEPSC amplitudes plotted in cumulative distribution functions (cdf). Control purple and DE maroon for IB (A-C) and RS cells (D-F). **A-C:** mEPSC amplitudes of IB neurons show increases in mEPSC amplitudes after 3d and 5d DE. **D-F:** mEPSC amplitudes of RS neurons show reduction in mEPSC amplitudes after 12hr of DE recovering to control values by 3d DE. Bin interval-1pA.

G-L: mEPSC inter-event intervals plotted in cumulative distribution functions (cdf). Control blue and DE green for IB (G-I) and RS cells (J-L). **G-I: -** inter-event intervals of mEPSCs in IB neurons do not show any significant change with DE. **J-L: -** inter-event intervals appear to increase at all the time points for RS cells but the difference is significant only at 12 hours and 5d DE. Bin interval-0.5sec. *$p<0.05$, ** $p<0.01$. Plotted points are means ± SEM.

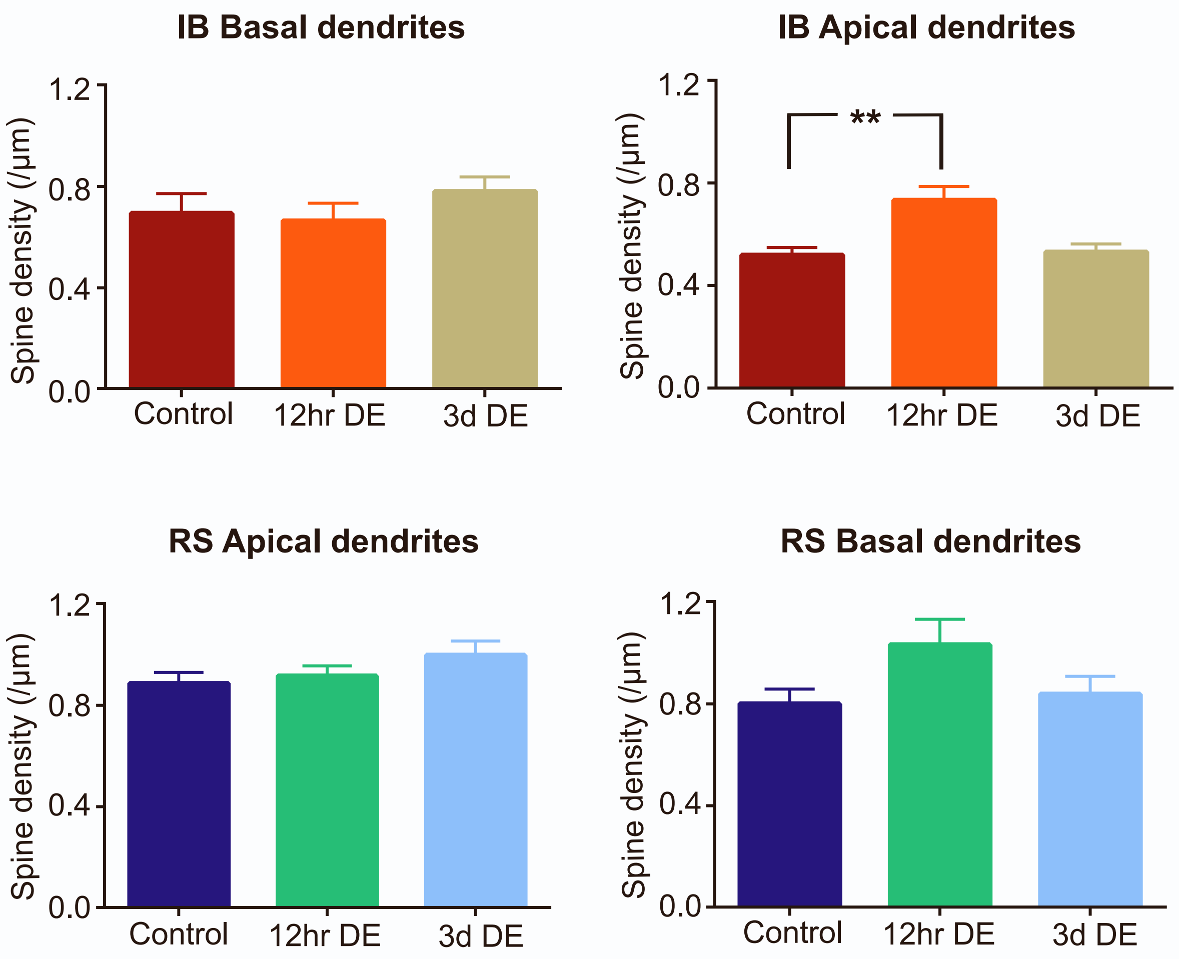

Figure S5. Effect of dark exposure (DE) on spine density (related to Figure 6).

A. DE does not change spine density on basal dendrites of IB neurons. **B.** On IB neurons' apical dendrites spine density increases with 12hr DE and comes back to baseline at 3d DE. In RS neurons DE has no significant impact on apical dendrites (**C**), or basal dendrites (**D**). ** $p<0.01$. Histograms represent means $\pm$ SEM.

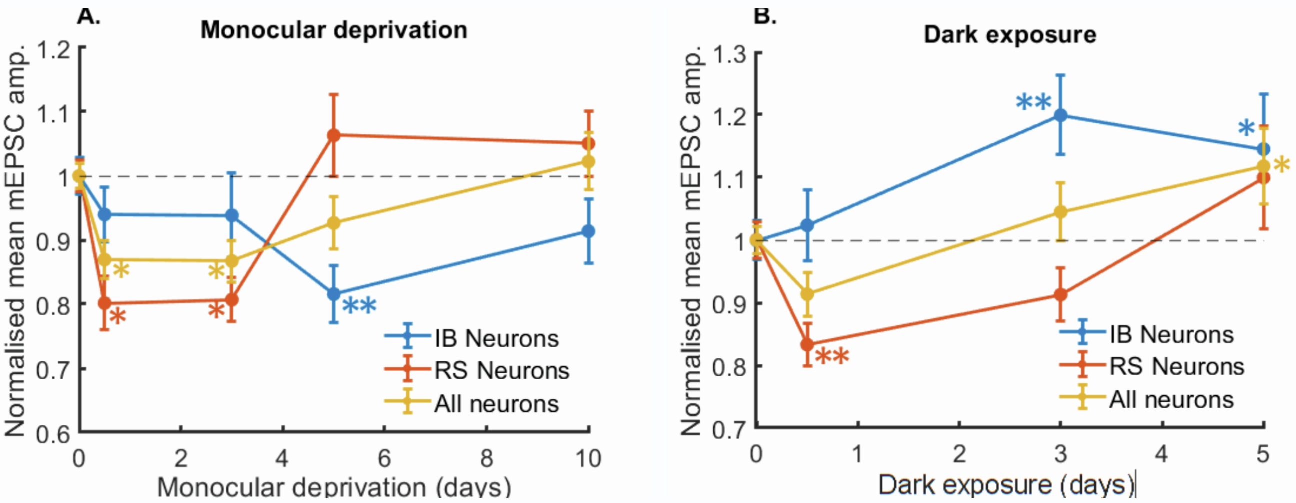

Figure S6. IB and RS data pooled together for MD and DE (related to Figures 3 and 4).

A: Layer 5 pyramidal neurons show Monocular Deprivation (MD) induced synaptic depression and a slow recovery toward baseline if data from IB and RS neurons are not treated separately (All neurons, yellow line and symbols). Note that IB (blue line and symbols) and RS neurons' responses (red line and symbols) exhibit different responses to MD, both from each other and from the pooled data.

B. Dark Exposure (DE) leads to small insignificant synaptic depression followed by late potentiation if the IB and RS neurons are not treated separately. The opposite direction of changes for RS and IB neurones largely cancel one another out. RS neurons (red line and symbols) only show depression and recovery, while IB neurons (blue line and symbols) only show potentiation due to DE. *$p<0.05$, ** $p<0.01$. Plotted points are means ± SEM.

AGE (days)	IB average IEI (s)	IB cell count	RS average IEI (s)	RS cell Count
26		0	1.207	2
27	0.151	1	0.925	6
28	1.248	8	0.738	11
29	0.647	7	0.573	3
30	0.850	2	0.913	5
31	0.797	3	1.039	1
32	0.952	5	1.349	3
33	1.190	11	1.217	10
34	0.946	10	1.558	10
35	0.623	2	1.516	6
36	0.739	3	0.794	5
38	0.729	1		0
TOTAL		**53**		**62**

Table S1. Analysis of changes in mEPSC inter-event interval as a function of age (related to Figure 2).

The median inter-event intervals for each cell are averaged for each age group and given in seconds. We found little correlation between age and inter-event interval over this period of development for Layer 5 RS (R^2 =0.18) and IB cells (R^2 = 0.01) and an ANOVA showed no significant effects (RS cells $F_{(1,61)}$=3.5, p=0.068; IB cells $F_{(1,52)}$=0.02, p=0.88).

Group	IB Animals	IB Neurones	RS Animals	RS Neurones	Age range (Days)
**Young undeprived**	19	27	16	31	27-32
**Old undeprived**	21	27	16	33	33-38
**IB undeprived (all data)**	40	54	--	--	27-38
**MD 12 hour**	7	9	7	9	27-30
**MD 3 day**	5	8	10	13	27-30
**Control for MD 5 day**	-	-	15	23	32-34 (RS)
**MD 5 day**	10	13	10	14	33-34 (RS), 31-34 (IB)
**Control for MD 10 day**	--	--	10	14	35-38 (RS)
**MD 10 day**	10	13	10	14	35-40 (RS), 34-40 (IB)
**DE 12 hour**	9	11	10	15	27-31 (RS), 27-31 (IB)
**DE 3 day**	9	12	12	14	28-31 (RS), 28-31 (IB)
**DE 5 day**	7	10	8	15	33-34 (RS), 33-34 (IB)
**XPro control (12hr & 3d DE)**	9	12	12	17	29-33 (RS), 28-33 (IB)
**XPro 12 hour DE**	8	13	9	13	28-34 (RS), 28-34 (IB)
**XPro 3 day DE**	7	8	7	13	29-32 (RS), 29-32 (IB)
**XPro control (for 5 day DE)**	6	8	6	10	31-33 (RS), 31-33 (IB)
**XPro 5 day DE**	6	8	8	11	31-33 (RS), 31-33 (IB)
**T286 control**	11	18	10	16	28-34 (RS), 28-34 (IB)
**T286 12 hour DE**	7	11	8	13	28-32 (RS), 28-31 (IB)
**T286 3 day DE**	6	12	5	9	30-33 (RS), 30-33 (IB)
**T286 5 day DE**	6	10	7	11	32-34 (RS), 32-34 (IB)
**TOTALS**	203	284	196	308	

Table S2. Number of animals and neurones in each subgroup (related to Star Methods). Note that MD = monocular deprivation, DE = dark exposure, XPro = treatment with XPro1595 and T286 = αCaMKII^{T286A} point mutant mice.
